# A Biobank of Breast Cancer Explants with Preserved Intra-tumor Heterogeneity to Screen Anticancer Compounds

**DOI:** 10.1016/j.cell.2016.08.041

**Published:** 2016-09-22

**Authors:** Alejandra Bruna, Oscar M. Rueda, Wendy Greenwood, Ankita Sati Batra, Maurizio Callari, Rajbir Nath Batra, Katherine Pogrebniak, Jose Sandoval, John W. Cassidy, Ana Tufegdzic-Vidakovic, Stephen-John Sammut, Linda Jones, Elena Provenzano, Richard Baird, Peter Eirew, James Hadfield, Matthew Eldridge, Anne McLaren-Douglas, Andrew Barthorpe, Howard Lightfoot, Mark J. O’Connor, Joe Gray, Javier Cortes, Jose Baselga, Elisabetta Marangoni, Alana L. Welm, Samuel Aparicio, Violeta Serra, Mathew J. Garnett, Carlos Caldas

**Affiliations:** 1 Department of Oncology and Cancer Research UK Cambridge Institute, Li Ka Shing Centre, University of Cambridge, Cambridge CB2 0RE, UK; 2 Cambridge Breast Unit, NIHR Cambridge Biomedical Research Centre and Cambridge Experimental Cancer Medicine Centre at Cambridge University Hospitals NHS Foundation Trust, Cambridge CB2 2QQ, UK; 3 Department of Molecular Oncology, British Columbia Cancer Research Centre, Vancouver, BC V5Z 1L3, Canada; 4 Wellcome Trust Sanger Institute, Wellcome Trust Genome Campus, Hinxton, Cambridgeshire CB10 1SA, UK; 5 DNA Damage Response Biology Area, Oncology IMED, AstraZeneca, Alderley Park, Macclesfield SK10 4TG, UK; 6 OHSU Knight Cancer Institute, Oregon Health & Science University, 3181 SW Sam Jackson Park Road, Portland, OR 97239, USA; 7 Vall d’Hebron Institute of Oncology, 08035 Barcelona, Spain; 8 Human Oncology and Pathogenesis Program, Department of Medicine, Memorial Sloan Kettering Cancer Center, NY 10065, USA; 9 Translational Research Department, Institut Curie, 26 rue d’Ulm, Paris 75005, France; 10 Huntsman Cancer Institute, Salt Lake City, UT 84112, USA

## Abstract

The inter- and intra-tumor heterogeneity of breast cancer needs to be adequately captured in pre-clinical models. We have created a large collection of breast cancer patient-derived tumor xenografts (PDTXs), in which the morphological and molecular characteristics of the originating tumor are preserved through passaging in the mouse. An integrated platform combining in vivo maintenance of these PDTXs along with short-term cultures of PDTX-derived tumor cells (PDTCs) was optimized. Remarkably, the intra-tumor genomic clonal architecture present in the originating breast cancers was mostly preserved upon serial passaging in xenografts and in short-term cultured PDTCs. We assessed drug responses in PDTCs on a high-throughput platform and validated several ex vivo responses in vivo. The biobank represents a powerful resource for pre-clinical breast cancer pharmacogenomic studies (http://caldaslab.cruk.cam.ac.uk/bcape), including identification of biomarkers of response or resistance.

## Highlights

• We developed a biobank of breast cancer patient-derived tumor xenografts (PDTXs)
• PDTXs represent diverse molecular subtypes and retain intra-tumor heterogeneity
• PDTX-derived tumor cells (PDTCs) were used for high-throughput drug testing
• PDTXs/PDTCs are a robust platform for pre-clinical pharmacogenomic studies

## Graphical Abstract

**Figure:**
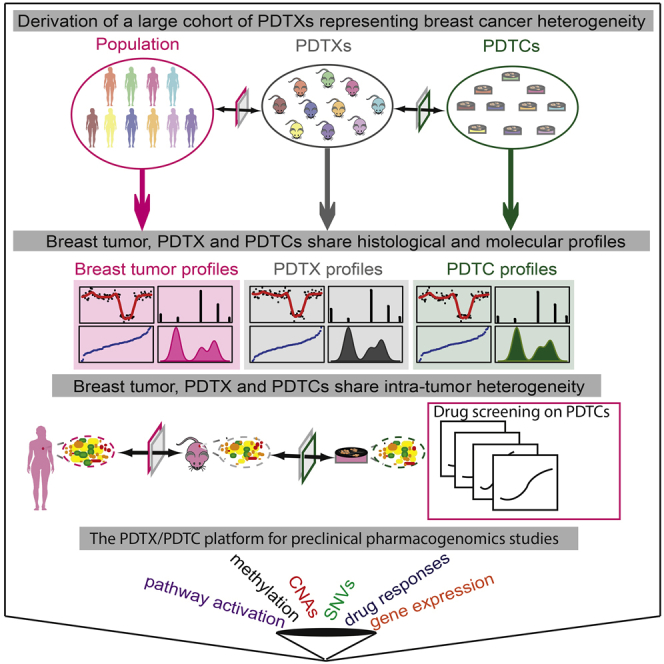

## Teaser

Development and analysis of a collection of breast-cancer-patient-derived xenografts indicate that the xenografts and cell cultures derived from them preserve the heterogeneity of the original tumors and can be used for drug screening.

## Introduction

Molecular stratification is the first step toward precision cancer medicine (Aparicio and Caldas, 2013). Recently, we reported (Curtis et al., 2012, Dawson et al., 2013, Dvinge et al., 2013) and validated (Ali et al., 2014) a genome driver-based molecular taxonomy of breast cancer. Modeling this diverse inter-tumor heterogeneity of breast cancer is challenging and requires generation of explant models representing the ten identified integrative clusters (IntClust).

Cancer cell lines have been extensively used for drug development and biomarker discovery (Heiser et al., 2012) but are successful at predicting clinical responses in only a handful of examples (Kim et al., 2015, Sharma et al., 2010). The modest clinical predictive value of cancer cell lines results from their recognized shortcomings: limited capacity to recapitulate inter- and intra-tumor heterogeneity and adaptation to growth in artificial conditions. These limitations are significant because both tumor subtype and cancer genome evolution, resulting in intra-tumor heterogeneity, remain the main challenges to successful cancer treatment.

The increasing understanding of cancer biology has led to the availability of targeted therapies. These drugs typically explore oncogene addiction or synthetic lethality (Kaelin, 2005, Luo et al., 2009, Torti and Trusolino, 2011). Unfortunately, the inherent heterogeneity of cancer means that either primary or acquired resistance nearly always occurs. Successful early drug development hence requires molecular stratification and characterization of intra-tumor heterogeneity.

Patient-derived tumor xenografts (PDTXs) have emerged as powerful pre-clinical models to recapitulate the diversity of human tumors (Cassidy et al., 2015). The greatest promise of PDTXs is their potential to improve the rates of attrition in cancer drug development (Aparicio et al., 2015, Gao et al., 2015, Hidalgo et al., 2014, Tentler et al., 2012). However, generalized use of PDTXs in high-throughput drug studies is unrealistic, for both cost and animal welfare reasons. Moreover, it has not been clear whether PDTXs retain the heterogeneity of the original tumor. Here, we demonstrate molecularly characterized PDTXs and their matched PDTX-derived tumor cells (PDTCs) in short-term culture do retain this heterogeneity and may be used as a platform for cancer drug screening with the potential to uncover molecular mechanisms of therapy response.

## Results

### Generation of Breast Cancer PDTXs Representing Most Breast Cancer Clinical and Molecular Subtypes

**Figure 1:**
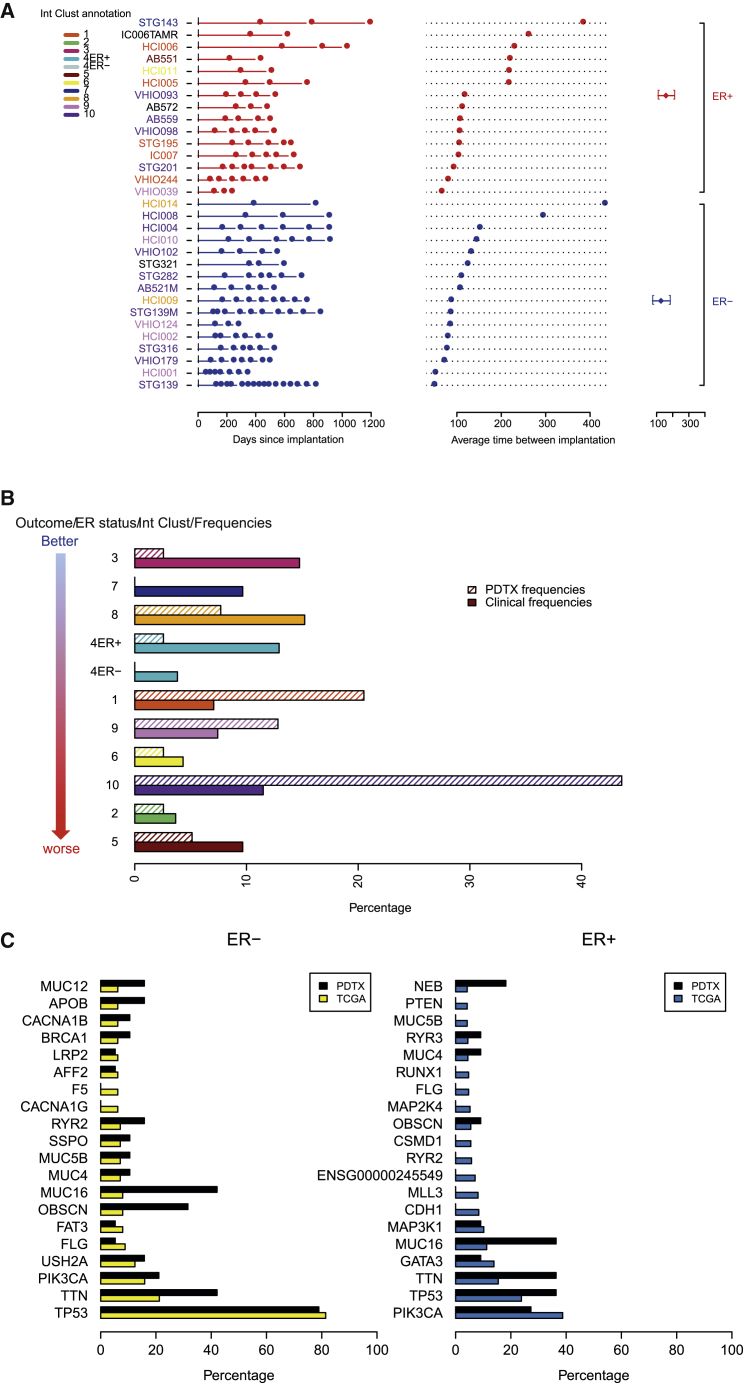
Derivation of an Extensively Annotated Breast Cancer PDTX-PDTC Biobank Representing Breast Cancer Subtypes (A) Timeline of engraftment for established PDTX models (n = 31; ER+ in red; ER− in blue). Each square represents a time point of engraftment. Average ER+ and ER− re-implantation time is shown on furthermost right panel. Model IDs are color coded according to integrative cluster (IntClust). (B) Bar plots showing the IntClust distribution of PDTX models (n = 40; shadowed) and for comparison primary breast cancers from METABRIC (n = 1,980; dense). (C) Distribution of somatic mutations in tumors from the TCGA cohort (n = 495) and PDTX models (n = 30), stratified by ER status. See also Figure S1.

We have established a large bank (n = 83) of live human breast cancer explants by implantation of tumor samples in highly immunodeficient mice (NOD.Cg-Prkdcscid Il2rgtm1Wjl/SzJ or NSGs; see STAR Methods). Comprehensive clinical information on the patients and originating cancer sample implanted to generate PDTXs can be found in Table S1. To date, PDTXs have been successfully established from both primary (n = 46) and metastatic (n = 37) sites, and more than 50% (n = 50) are from ER+ disease (Table S1). The PDTX growth rates upon initial engraftment and after subsequent re-implantation were variable across models, remained mostly stable upon serial engraftment, and tended to be faster in explants originated from ER− tumors (Figure 1A shows data for 31 models). Importantly, all established models tested to date could be flash frozen and subsequently successfully engrafted, ensuring the persistence of the living biobank.

**Figure S1:**
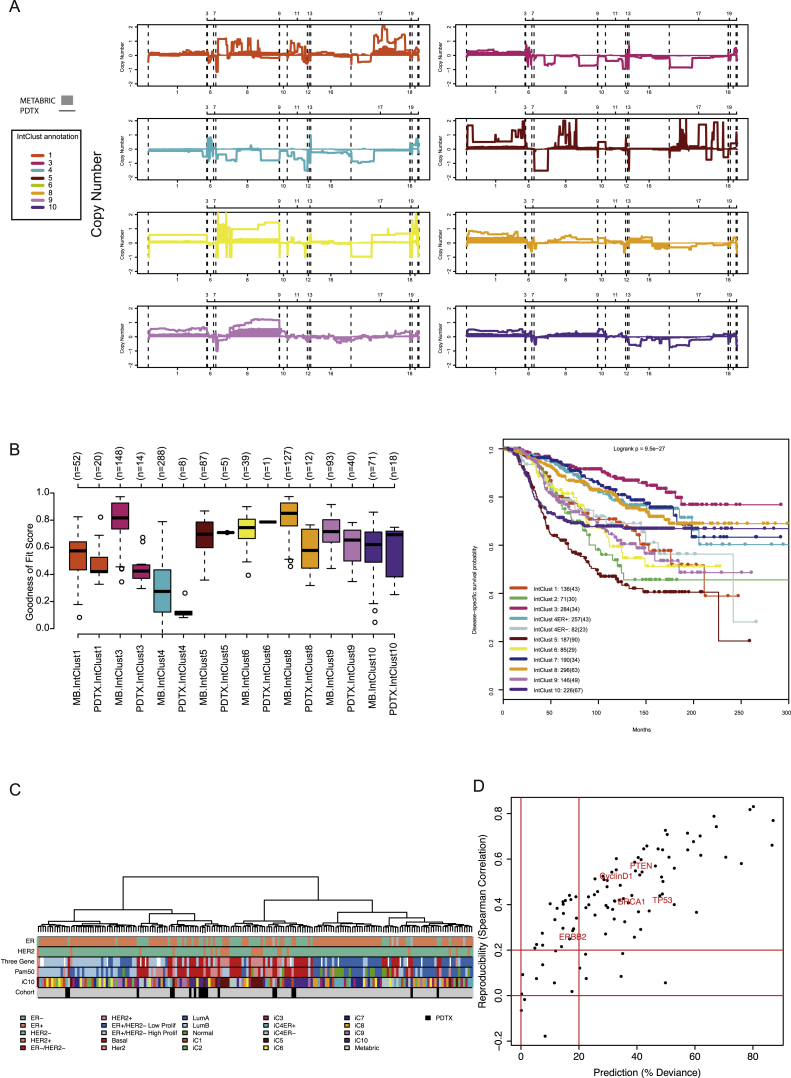
Related to Figure 1 and STAR Methods (A) Copy number profiles based on the *cis*-features that define the 10 Integrative Clusters (Curtis et al., 2012) in the METABRIC discovery dataset (n = 997 breast tumors) and the PDTX biobank (n = 121 samples from 36 models). (B) Left panel: Goodness of fit scores for copy number-based classification into IntClust subtypes (Metabric cohort n = 905 tumors, PDTX cohort n = 87 samples from 36 models). Right panel: Kaplan-Meier (disease-specific survival) of IntClust subtypes for the METABRIC cohort. IntClust4 was further stratified into ER-+ and ER-. (C) Hierarchical clustering (Ward’s method) of cancer pathway activation scores across samples. Included are 15 randomly picked samples belonging to each of the 10 IntClusts from the METABRIC cohort and 19 PDTX models (plus 5 technical replicates). (D) Scatter plot of reproducibility and prediction accuracy of cancer pathway activation scores in PDTXs (n = 78 tumor/PDTX pairs from 16 models). Reproducibility was assessed using Spearman correlation of cancer pathway scores in matched tumors and PDTXs. Prediction accuracy was determined by fitting a generalized additive model and computing the deviance explained.

In order to be classified into one of the IntClust using the method we described (Ali et al., 2014), the PDTXs were subject to copy number profiling, by shallow whole-genome sequencing (“sWGS”), and expression profiling, by microarrays (“RNAexp”). The copy number profiles of PDTXs classified into each IntClust were similar to those reported in primary tumors (Curtis et al., 2012; Figure S1A). The goodness of fit scores of IntClust assignment were computed (for IntClusts with more than one xenograft model: IntClust1; IntClust3; IntClust4; IntClust5; IntClust6; IntClust9; and IntClust10), and with one exception (IntClust3), these scores were very similar to classifying primary tumors (Figure S1B).

Copy number aberrations (CNAs) in known breast cancer driver genes (Curtis et al., 2012) present in the PDTXs included gains/amplifications of *MYC* (78%), *CCNE1* (34%), *ZNF703* (25%), *CCND1* (31%), *MDM2* (25%), and *ERBB2* (9%) and deletions of *PTEN* (41%), *PPP2R2A* (72%), *CDKN2A* (47%), and *CDKN2B* (47%). This CNA frequency distribution is different from that seen in a breast cancer clinical population (METABRIC dataset) and reflects both the origin of the PDTXs (around 45% were from metastatic biopsies) and the disproportionate engraftment of triple-negative basal-like cancers (IntClust10) and more-aggressive subtypes of ER+ tumors (IntClust1 and IntClust9; Figure 1B). In contrast, we observed lower engraftment of ER+ tumors from better-outcome subtypes (IntClust3, IntClust4, IntClust7, and IntClust8; Figure 1B; Table S1).

Downstream analysis of mRNA expression data using the gene set variation analysis (GSVA) approach (Hänzelmann et al., 2013), a method to estimate pathway activity, also showed that the diversity of activity scores in cancer-related pathways (Molecular Signatures Database; http://software.broadinstitute.org/gsea/msigdb; Liberzon et al., 2011) in PDTXs was similar to that observed in the breast cancer clinical population (Figure S1C). Furthermore, in matched pairs, the activity of breast-cancer-related pathways (e.g., PTEN, Tp53, BRCA1, Her2, and Cyclin D1) in the PDTXs was correlated with and predicted the activity scores in the originating breast cancer samples (Figure S1D).

The subtype distribution of engrafted PDTXs was also reflected by the mutation frequencies identified using whole-exome sequencing (“WES”). The most-commonly mutated genes in ER− breast cancers (Cancer Genome Atlas Network, 2012) were mutated at similar frequencies in ER− PDTXs (Figure 1C). In contrast, frequencies of mutated genes in ER+ PDTXs mirrored those found in more-aggressive subtypes of ER+ tumors (Pereira et al., 2016). As an example, *PIK3CA* mutations were found in only 27% of ER+ PDTX models (Figure 1C), compared to 46% and 38% in the METABRIC and The Cancer Genome Atlas (TCGA) cohorts, respectively.

In summary, these data show that we have successfully generated a living biobank of breast cancer xenografts, representing the clinical and molecular diversity of the disease.

### PDTXs Retain Their Original Histological and Molecular Features through Passaging

**Figure S2:**
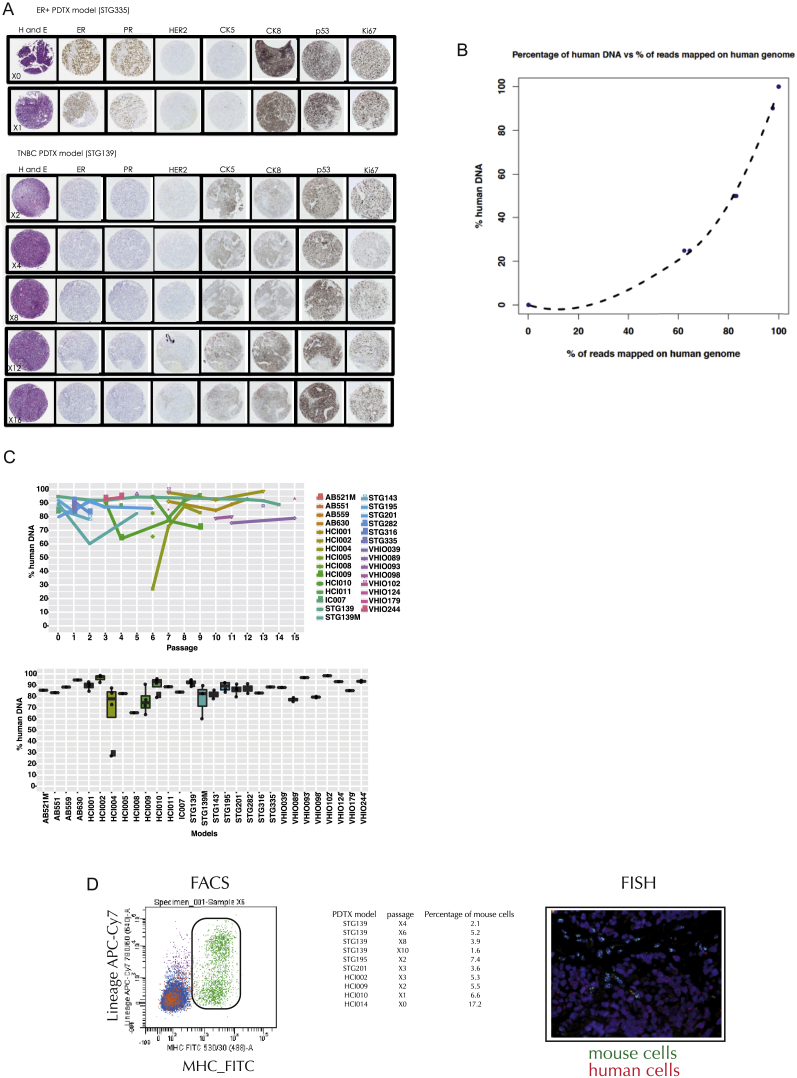
Related to STAR Methods and Figures 2A and 2B (A) Representative images from the histopathological analysis of PDTXs. Images from both an ER+ (STG335) and a triple negative (STG139) model at different passages are shown. (B) Calibration curve for estimation of mouse content based on the proportion of mapped reads from WES to the human genome. (C) Estimated percentage of mouse contamination from WES data. Top panel: data for each model at different passages is represented. Bottom panel: Box plots showing the distribution of percentage of human cells per model in all samples tested, including different mice from the same passage and the same model, and different passages of the same model (n = 94 samples from 29 models; estimates from the same model and passage have been averaged). (D) Representative FACS plot of single cells (left) and a FISH image (right) on an FFPE tissue section from an example PDTX sample. Table shows average percentage of mouse cells in different PDTX models tested by FACS.

Histologically, PDTXs (23 models analyzed) showed similar morphology to the originating tumor; tubule formation and associated stroma were present in the xenograft, as seen in the matched patient cancer sample (Figure S2A). Histological review of multiple PDTX passages (Table S2) revealed that tumor tissue morphology remained stable with serial engraftment. Analysis of immunohistochemistry for epithelial markers (CK5, CK8, CK14, CK18, E-cadherin, and epithelial specific antigen) and for clinical biomarkers (ER, PR, Her2, Ki67, and p53) showed these features were similar in matched pairs of PDTX model and originating breast cancer sample and were consistently retained with passaging (Figure S2A and Table S2 for summary of the data).

The PDTX samples were comprehensively molecularly characterized at several passages using sWGS (for CNAs), WES (for single nucleotide variations [SNVs]), reduced-representation bisulfite sequencing (“RRBS”) (for DNA methylation), and RNAexp (for global expression and pathway activity profiling).

The analysis of sequencing data from PDTX samples is complicated by the presence of a variable and unknown amount of mouse cells. To address this, a serial dilution series of control samples with known mixtures of human and mouse DNA was created to develop a robust computational pipeline to discriminate human and mouse reads with an accuracy >99.9% (see Figure S2B and STAR Methods for details). This pipeline identified three spontaneous mouse tumors arising at or near the implantation site, which were discarded from further experiments. Post-filtered aligned data from this pipeline were used for somatic copy number and mutation calling (see STAR Methods).

**Figure 2:**
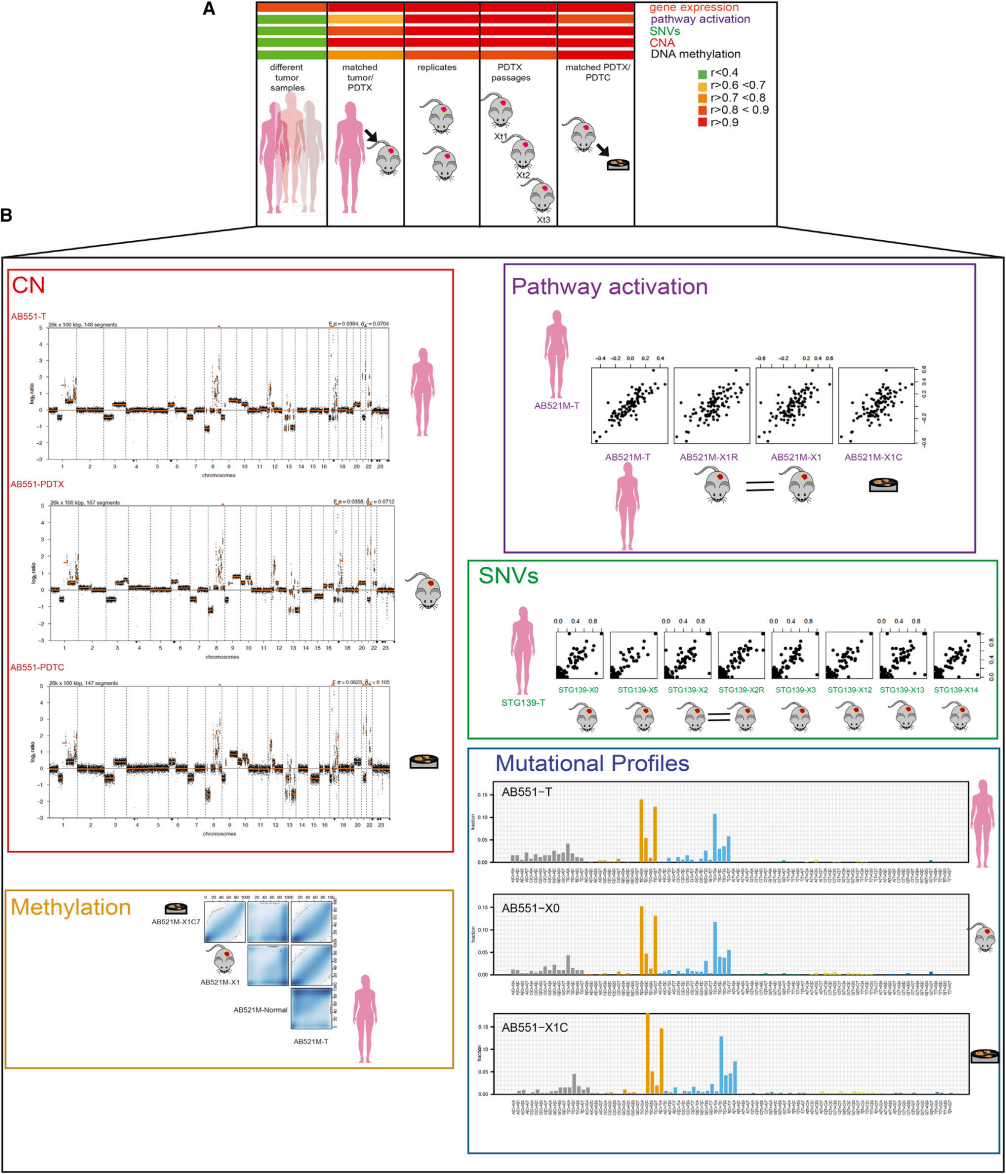
PDTXs Closely Match Originating Patient Cancer Samples (A) Heatmap of Pearson correlation scores across molecular data types (different sample sizes described in the main text). (B) Panels with individual examples for five types of molecular data. (Left panel: top) CNA plots for AB551 (originating sample [T], PDTX, and PDTC) are shown; (bottom) scatterplot of methylated CpGs (from RRBS data) in AB521M is shown. (Right panel: top) Scatterplots of pathway activity scores in AB521M are shown. (Middle) Scatterplots of variant allelic fractions in STG139 are shown. (Bottom) Mutational profiles in AB551 are shown.

We used these data to determine how implantation, serial passaging, and replicate engraftment affected gene expression, cancer pathway activation scores, allelic fractions of somatic mutations, CNAs, and DNA methylation. This analysis, done also for comparison in reference sets (different tumor samples and technical and biological replicates), revealed a high degree of correlation in matched sample pairs for all data types (Figure 2A).

The biological relevance of the models was evidenced by similar gene expression profiles between the originating tumor and the PDTX (RNAexp n = 44; r = 0.95; interquartile range [IQR] 0.94–0.97), oncogenic pathway scores (r = 0.67; IQR 0.44–0.79), CNA profiles (sWGS n = 45; r = 0.91; IQR 0.84–0.93), SNV allelic fractions (WES n = 82; r = 0.81; IQR 0.74–0.88), and DNA methylation profiles (RRBS n = 8; r = 0.82; IQR 0.79–0.84). The biological robustness of the models across serial passaging was also remarkable, with retention of gene expression profiles (RNAexp n = 217; r = 0.98; IQR 0.97–0.99), oncogenic pathway scores (r = 0.87; IQR 0.80–0.93), CNA profiles (sWGS n = 109; r = 0.97; IQR 0.92–0.98), SNV allelic fractions (WES n = 201; r = 0.92; IQR 0.87–0.95), and DNA methylation profiles (RRBS n = 37; r = 0.82; IQR 0.78–0.90; Figure 2A). Mutational signatures (Alexandrov et al., 2013, Rosenthal et al., 2016) in matched PDTXs across serial passages and the originating sample were also conserved (Figure 2B). Representative examples across the molecular data types for individual PDTX models are shown in Figure 2B.

In summary, the comprehensive characterization of histopathological characteristics, somatic genomic aberrations (CNAs and SNVs), methylation profiles, and gene expression of the biobank of human breast cancer explants confirms these models have a remarkable level of multi-dimensional molecular resemblance with their matched cancer of origin, significantly extending the observations we and others had previously reported (DeRose et al., 2011, Eirew et al., 2015, Li et al., 2013, Marangoni et al., 2007). Our findings robustly demonstrate these multi-dimensional molecular features are conserved through serial engraftment in the mouse.

### Mouse Stromal Composition of PDTXs Remains Stable through Passaging

Breast cancer PDTXs retain similar architecture to the originating tumor and, through passaging, this remains stable. This occurs despite mouse stroma replacing the human stroma (DeRose et al., 2011, Hidalgo et al., 2014). We used the custom sequencing analysis pipeline described above (Figure S2B; STAR Methods) to deconvolute the proportion of mouse DNA sequences in PDTX samples as a surrogate for mouse stromal cell content. Out of the 94 xenograft samples examined, only five had more than 40% mouse cells. Replicates obtained from these five had lower mouse stromal content, reflecting intra-PDTX heterogeneity (Figure S2C). The data from multiple PDTX models also showed the proportion of mouse content does not change significantly across passages (Figure S2C). Two independent methods were used to validate these observations: fluorescence-activated cell sorting (FACS) of PDTX-derived single-cell suspensions with an MHC-class I anti-mouse H-2Kb/H-2Db antibody and fluorescence in situ hybridization (FISH) with mouse and human centromeric probes in tissue sections (Lawson et al., 2015, Li et al., 2013; Figure S2D).

In summary, these data show the mouse stroma contribution to the xenografts is stable across serial passaging.

### Intra-tumor Heterogeneity and Clonal Architecture Are Maintained in PDTXs

Human breast cancers are composed of clones differing in mutation content (Aparicio and Caldas, 2013), resulting in intra-tumor genomic heterogeneity. This intra-tumor heterogeneity, although variable across tumors, is already present at diagnosis (Shah et al., 2012) and evolves dynamically in space and time (Ding et al., 2010, Murtaza et al., 2015, Shah et al., 2009).

WES data were used to interrogate both intra-tumor heterogeneity and clonal architecture in matched originating tumor, initial engrafted, and serially passaged xenografts.

**Figure S3:**
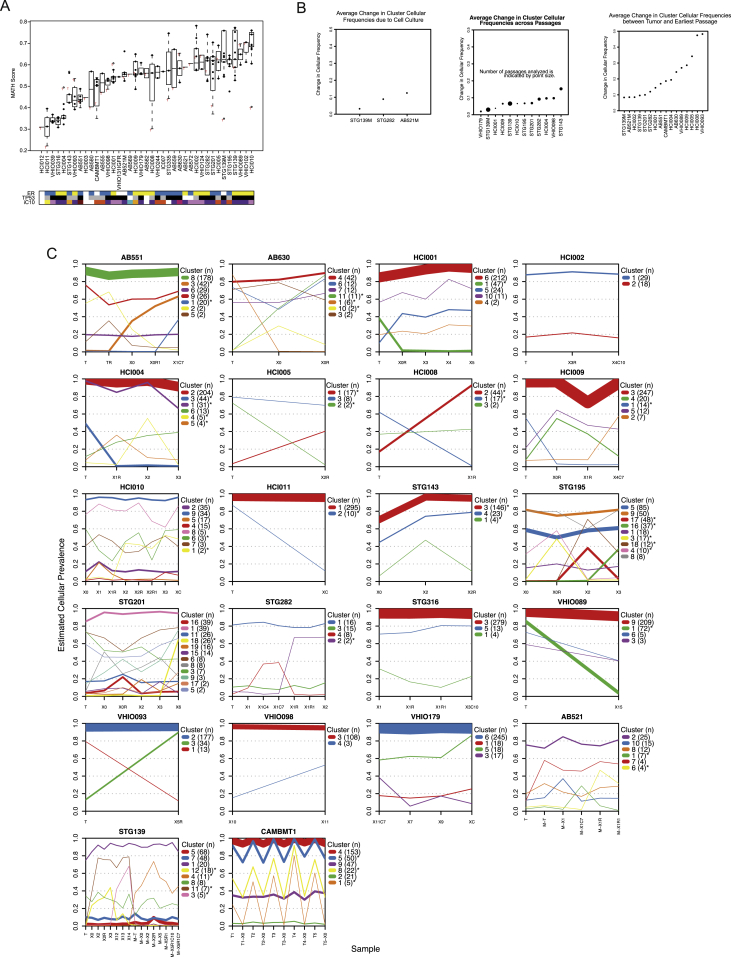
Related to Figure 3 and STAR Methods (A) Box plots of MATH scores for each model analyzed (n = 238 samples from 39 models). Each box plot represents the distribution of scores for matched tumor (red T), PDTXs and PDTCs. (B) Plots of average change in clonal cluster prevalence. PyClone was used to infer clonal architecture for the set of samples from each PDTX model. For all PDTX models with more than two samples, the absolute change in clonal cluster prevalence was averaged over all clusters. Left plot: average change in clonal cluster prevalence with short-term culture of PDTX cells (n = 5 comparisons from 3 models). Middle plot: average change in clonal cluster prevalence with serial passaging (n = 38 passages from 12 models). Right plot: average change in clonal cluster prevalence with implantation (originating sample versus earliest PDTX passage; n = 24 pairs from 16 models). Dot size is proportional to number of samples analyzed. (C) PyClone individual cluster plots showing clonal mean cellular prevalences for 22 models. Width lines are proportional to the number of variants in each clonal cluster. The legend indicates the name of the cluster and the number of variants in it. Asterisks remark clusters whose cellular frequencies are significantly different between samples. Only clusters with at least one variant are shown in the plot.

Quantification of intra-tumor heterogeneity using the mutant-allele tumor heterogeneity (MATH) method (Mroz and Rocco, 2013) revealed that the originating patient tumor samples had a range of scores (from low to high), as expected given their diverse IntClust subtype. The heterogeneity scores in multiple passages of matched PDTXs were similar, demonstrating explants preserve intra-tumor heterogeneity (Figure S3A).

**Figure 3:**
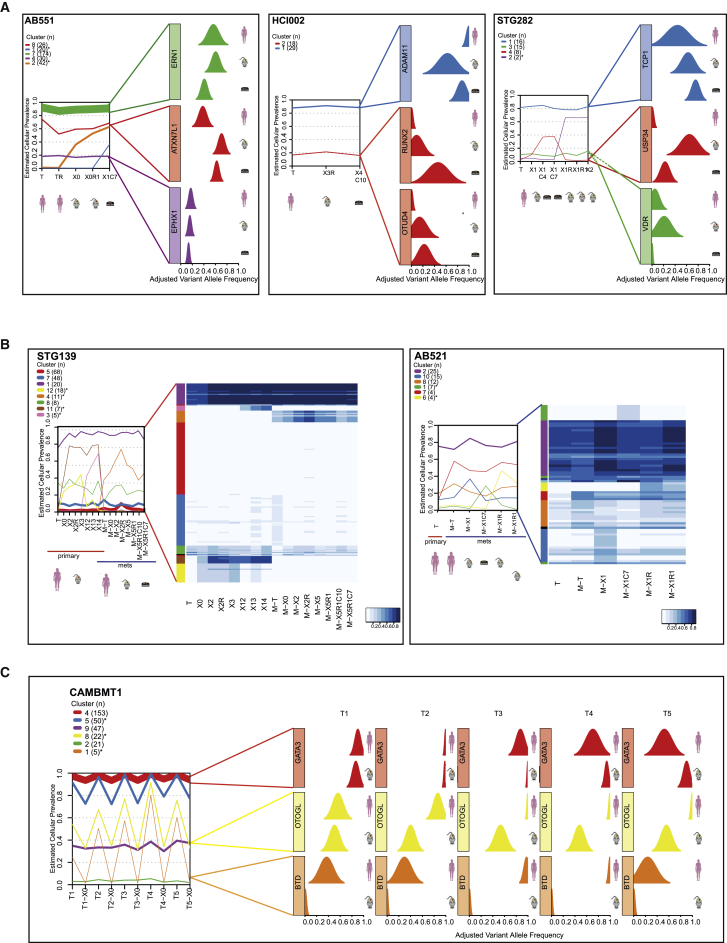
Clonal Architecture and Clonal Dynamics of Breast Cancer PDTXs (A) Example plots of AB551 (left panel), HCI002 (middle panel), and STG282 (right panel). (Left graph) The mean cellular prevalence estimates of mutation clusters in originating patient samples (T) and subsequent xenograft passages (Xn; n for passage number) or PDTCs (XnCy; y for days in culture) are shown. PyClone was used to infer clusters and cellular prevalence using WES data. Line widths indicate the number of SNVs comprising each mutation cluster (numbers in brackets adjacent to each plot). Asterisks indicate clonal clusters with significant changes in cellular prevalence. (Right graph) Plots of distribution of variant allele frequency for selected genes within clusters. (B) PyClone plots (as in A) and cellular prevalence heatmap plots for STG139 and AB521 samples. (C) PyClone plot and plots of distribution of variant allele frequency for selected genes within clusters (as in A) of five spatially separated biopsies and their matched xenografts in CAMBMT1. See also Figure S3.

Clonal architecture in individual samples and clonal dynamics upon engraftment and across serial passaging were assessed on 104 samples from 22 models using PyClone (Roth et al., 2014), as we recently described (Eirew et al., 2015). PyClone identified 190 clonal clusters across the samples analyzed, but only 38 clonal clusters (20%) had significant changes in cellular prevalence estimates (Table S3 for extended information from PyClone analysis in all models tested). Clonal selection was seen upon initial engraftment (average change in clonal prevalence 0.21) but minimal through serial transplantation (average change in clonal prevalence 0.07; Figure S3B). We next asked whether clonal clusters showing engraftment-associated dynamics were enriched for cancer drivers. Recently, our group used a ratiometric method (Vogelstein et al., 2013) to identify 40 breast cancer mutation driver genes in 2,433 breast cancers (Pereira et al., 2016). Remarkably, in only 4 of the 38 clonal clusters that changed significantly after engraftment or during passaging could we identify a mutation driver: *BAP1* in STG139 (cluster 12); *KDM6A* in HCI004 (cluster 3); *MAP3K1* in STG143 (cluster 3); and *PIK3CA* in HCI008 (cluster 2; Table S3). These data strongly suggest that most of the clonal dynamics within xenografts are not associated with known driver genes. Figure 3A shows examples both of individual clonal cluster plots and of variant allele frequency distributions for individual genes within these clusters. Figure S3C shows all individual clonal cluster plots generated from the 22 models analyzed to illustrate the full diversity of clonal architectures observed in the PDTX biobank.

We analyzed in detail the clonal architecture of two cases for which we had both primary and subsequent metastasis samples: STG139 and a lung metastasis 12 months later, STG139M, and AB521 and a liver metastasis 8 months later, AB521M. For the first patient, we generated a xenograft from the primary tumor (STG139-X) and a xenograft from the lung metastasis (STG139M-X), and for the second patient, we generated a xenograft from the liver metastasis (AB521M-X). Figure 3B shows the clonal cluster plots and a heatmap of variant allele frequencies derived from WES data for sample sets from both patients. Clonal clusters shared by both the originating primary tumor and metastatic samples (STG139: clonal clusters 1, 7, and 8; AB521: clonal clusters 2, 8, and 10) had stable cellular prevalence across passaging. These clusters contained around 80% of all SNVs detected in these two cases (Figure 3B) and included not only the stem or truncal cluster but significantly also included sub-clonal and even very minor clusters (estimated cellular prevalence <5%). Metastasis-only clonal clusters (STG139: cluster 4; AB521: cluster 7) were also preserved upon serial passaging. Finally, there were clusters detected only in engrafted-derived samples (clonal clusters 3, 11, and 12 in STG139 and 6 in AB521, respectively). Although these results need a degree of cautious interpretation (only two cases analyzed), it demonstrates that both originating tumor and xenografts contain multiple clones, and the dynamics of clones in the patient (by comparing primary and metastasis biopsies) and in the mouse (by comparing passages) have both similarities and differences. Detailed analyses of clonal dynamics in more matched primary metastatic samples and their derived PDTXs, with mirrored treatment regimes, will be extremely informative toward understanding the mechanisms that are operative in tumor clonal ecosystems (Heppner, 1984, Tabassum and Polyak, 2015).

From one large breast cancer brain metastasis (CAMBMT1), we obtained five spatially separate biopsies, which were implanted into five different NSG mice. WES data from all five biopsies showed similar clonal architectures (Figure 3C, left panel; Table S3). This case allowed us to compare the clonal architecture of the five xenografted samples, revealing remarkable similarity, despite some variation in the originating cellular prevalence in the separate biopsies (see, for example, variant allele frequencies of *GATA3*, *OTOGL*, and *BTD*; Figure 3C, right panel). These near identical clonal dynamics upon engraftment strongly suggest deterministic mechanisms operate on clonal selection and validate our previous hypothesis that specific mutations act as genetic markers of fitness and dictate evolutionary trajectories (Eirew et al., 2015).

In summary, these data show PDTXs constitute a pre-clinical model that captures the most-clinically relevant feature in human cancer: heterogeneous genomic architecture that dynamically evolves. Moreover, the data also indicate that the clonal dynamics of the derived and serially passaged explants are not stochastic.

### Generation of Short-Term Cultures of PDTCs

**Figure S4:**
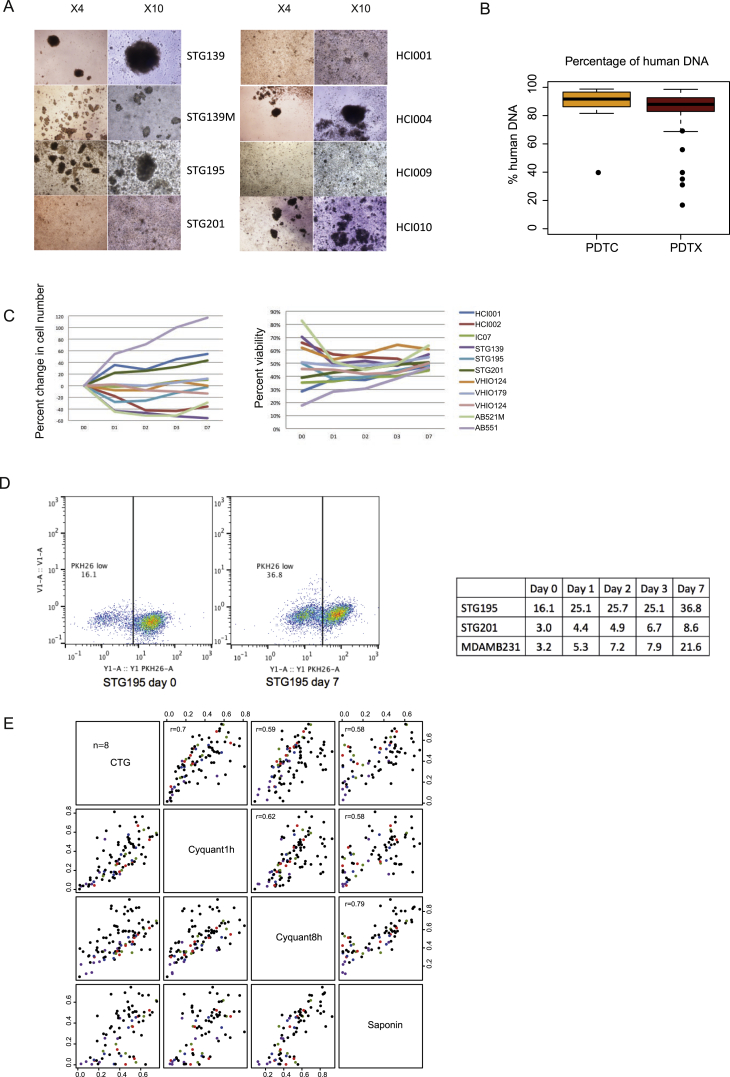
Related to Figure 4 and STAR Methods (A) Representative microscopy images from 8 PDTX models of short-term cultures (PDTCs) at day 7 after plating. (B) Box plots showing the percentage of human DNA on PDTX (n = 94 samples) and PDTC (n = 15 samples) models (n = 29). Data estimated from our sequencing-based approach. (C) Changes in cell number and viability of PDTCs at each time-point. (D) Representative FACS image from PKH26 assay. Table showing quantification of PKH26 low cells in 2 PDTCs (STG195 and STG201) and a highly proliferative breast cancer cell line (MDAMB231), shown for comparison purposes, at different time-points. (E) Scatterplots comparing AUC values measuring drug response for 19 drugs in 8 models (AB521, AB555, AB582, STG195, STG316, STG321, STG335 and VHIO093) using 3 different viability assays. 10 different doses were tested. Curve fitting and computation of the AUC was done as described in the STAR Methods. Dots highlighted in color correspond to PI3K pathway inhibitors: GDC032 (PI3Kα) in red, GDC0941 (pan PI3K) in blue, AZD6482 (PI3Kβ) in purple, and AZD8055 (mTOR) in green.

The PDTXs described constitute a living biobank of breast cancer explants that retain through passaging the inter- and intra-tumor heterogeneity encountered in the clinic. We therefore developed a method to enable the use of this valuable resource for high-content pre-clinical drug screening, similar to the approach widely used with cell lines (Barretina et al., 2012, Garnett et al., 2012). The method involved optimizing short ex vivo culture of cells isolated from the PDTXs (named PDTCs). These short-term PDTC cultures were successfully generated from all models where attempted (n = 27, at least two different passages from each; Figure S4A; see STAR Methods).

Sequencing data confirmed that the PDTCs had a proportion of mouse-derived cells similar to that found in the originating PDTX (Figure S4B). Cell proliferation, cell viability, and cell divisions (measured by PKH26 assay) were analyzed in the cultures and showed the expected variability across models, reflecting the diversity of the originating cancer (Figures S4C and S4D).

PDTCs derived from ten of the PDTX models were extensively characterized using WES, sWGS, and RNAexp. Analysis of these data showed the short ex-vivo-cultured PDTCs retained the molecular features of the originating PDTX (Figures 2A and 2B), including similar clonal architecture (Figures 3A, S3B, and S3C; Table S3). The average absolute change in clonal cluster cellular prevalence in matched PDTC-PDTX pairs was 0.08 (Figure S3B, left panel).

In summary, PDTCs can be systematically and consistently generated from PDTXs and retain their genomic features, making them an excellent model system for high-throughput drug screens.

### High-Throughput Drug Screening in PDTC Models

We tested the use of PDTCs as a pre-clinical drug-screening platform with an approach similar to that which we previously reported for cell lines and organoids (Garnett et al., 2012, van de Wetering et al., 2015). A selection of 22 different PDTX models were plated as PDTCs and 24 hr later screened with 108 compounds, representing a total of 6,634 drug tests performed (see STAR Methods). The compounds used were either approved cancer treatments or drugs targeting key cancer pathways (Table S4). The effect of drug treatment on cell viability was determined by CellTiter-Glo (CTG) (Garnett et al., 2012, van de Wetering et al., 2015) and drug responses represented by (1) the half-maximal inhibitory concentration (IC_50_), (2) the dose-response curve, and (3) the area under the dose response curve (AUC). In total, 2,550 drug-PDTC combinations were tested, with a range of 5–20 (mean = 16) PDTC models screened per drug. For most models, the drug treatment was performed in at least three technical replicates (same model, same passage, and same mouse) and in two or three biological replicates (same model, different passage).

One significant limitation of these analyses is that these measurements (IC_50_ and AUC) did not account for cell division rates across the different PDTC models. Growth rate inhibition metrics have recently been shown to provide more-reliable measurements of sensitivity to cancer drugs (Hafner et al., 2016). Nevertheless, we have been able to make several observations that attest to the value of the drug screening results obtained despite this caveat.

**Figure 4:**
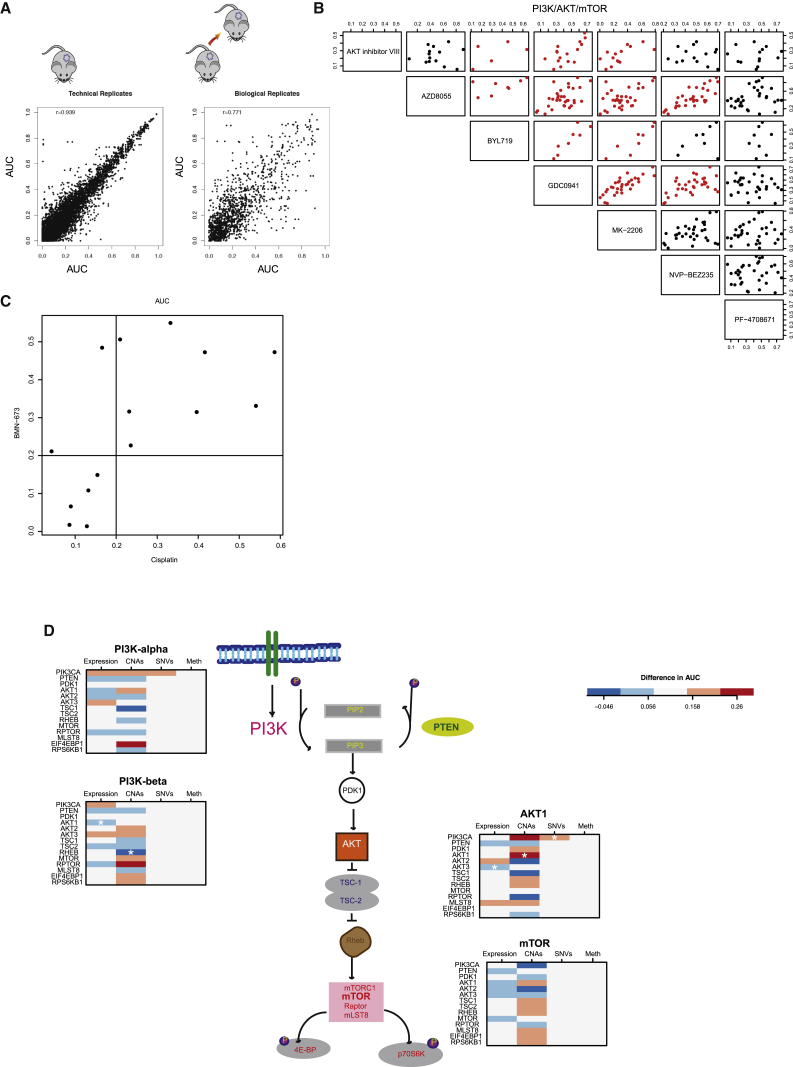
High-Throughput Drug Screening Using PDTCs (A) AUCs scatterplots showing reproducibility of PDTC drug testing. (Left panel) AUCs of technical replicates (n = 6,325; same sample, same compound) are shown. (Right plot) AUCs of biological replicates (n = 1,341; same model, different passages, same compound) are shown. r, Pearson correlation. (B) AUC scatterplots of all drugs targeting PI3K/AKT/mTOR pathway (n = 34 passages from 20 models). Red indicates Pearson correlation > 0.5. (C) AUC scatterplot for cisplatin and BMN-673 treatment across models tested (n = 15). (D) Illustration of the PI3K pathway with panels depicting difference in the AUC in models (n = 15) with versus without molecular alteration in pathway member. (Left panels) Inhibitors of PI3K alpha and PI3Kbeta are shown. (Right panels) Inhibitors of AKT1 and mTOR are shown. See also Figures S4, S5, and S6.

First, the observed AUC values across all drugs and models tested were highly correlated across technical (Pearson correlation of 0.94) and biological replicates (Pearson correlation of 0.78; Figure 4A). These results are highly similar to those we previously reported in established cell lines or tumor organoids (Garnett et al., 2012, van de Wetering et al., 2015). To further verify the robustness of these in vitro drug response data, we tested in eight PDTC models a set of 19 drugs using CyQUANT and Sytox endpoint assays, in addition to CTG (see STAR Methods). The results of these experiments revealed highly correlated drug responses independently of the assay used (Figure S4E; Table S5).

Second, analysis of AUC data for compounds targeting the same pathway or with similar mechanism of action showed highly correlated response profiles. One example with inhibitors of the PI3K-AKT-mTOR pathway (NVP-BEZ235/dactolisib, AZD8055, GDC0941/pictilisib, AKT inhibitor, and MK-2206) is shown in Figure 4B. Another example with compounds targeting homologous recombination repair defects (PARP inhibitor BMN-673/talazoparib and cisplatin, a DNA cross-linking agent) is shown in Figure 4C.

**Figure S5:**
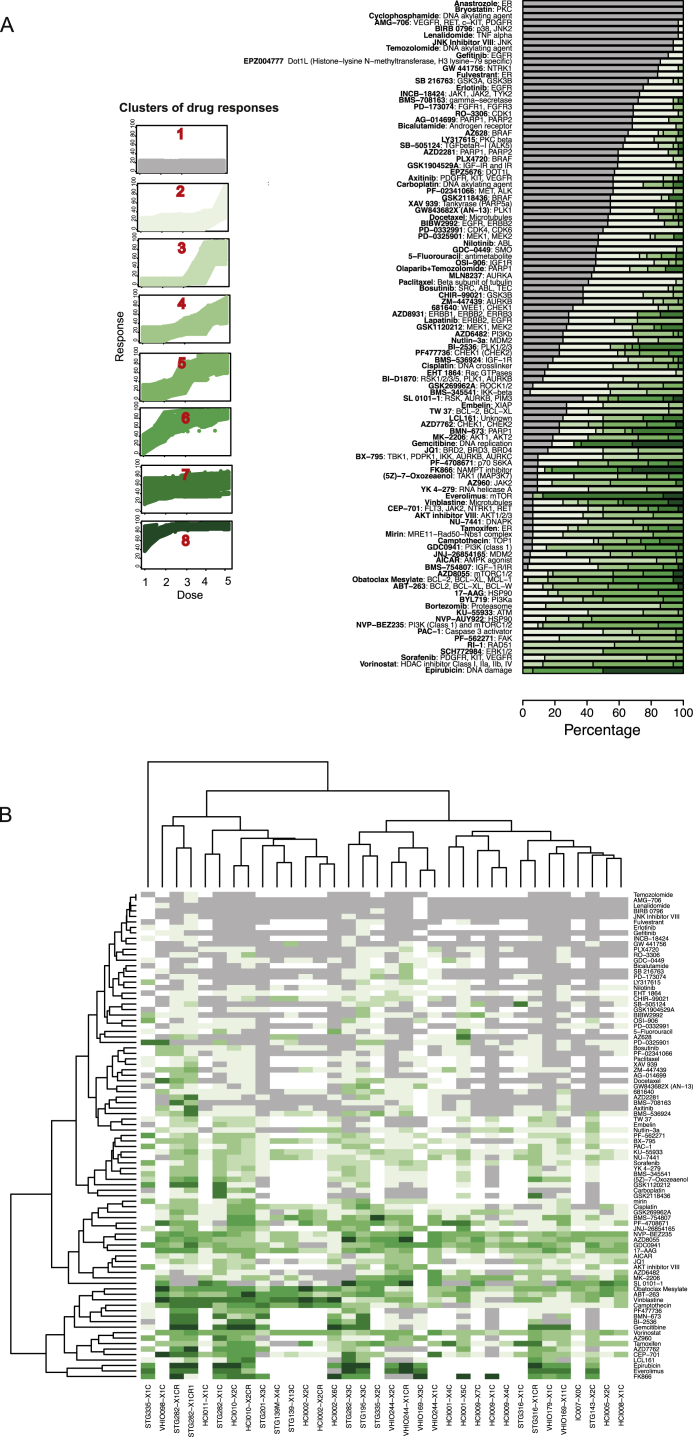
Related to Figure 4 and STAR Methods (A) Drug responses classified into 8 different groups according to response curves. Left panel- the drug responses clustered into 8 groups. Right panel- plots with percentage of samples (n = 37 samples from 20 models) displaying each drug response pattern for each compound. Drug name (bold) and putative target indicated. (B) Unsupervised clustering of drug responses in tested models (n = 37 samples from 20 models) according to subtype of drug response pattern (color coded as in A). Vertical axis- drugs; horizontal axis- models.

Distinct PDTC models can sometimes share the same IC_50_ and AUC values for a compound and have very different dose-response curves. Hence, a new method, based on the pattern of the slope of the dose-response curve, was developed to classify drug sensitivity patterns into eight groups (see STAR Methods). Figure S5A shows for each compound the proportion of drug responses classified into each of the eight drug sensitivity patterns across all models tested with that drug. Clustering of drug sensitivity patterns (Figure S5B) confirmed the high reproducibility and biological robustness of the data: different passages of the same model and compounds with similar mechanisms of action and target specificities clustered together.

**Figure S6:**
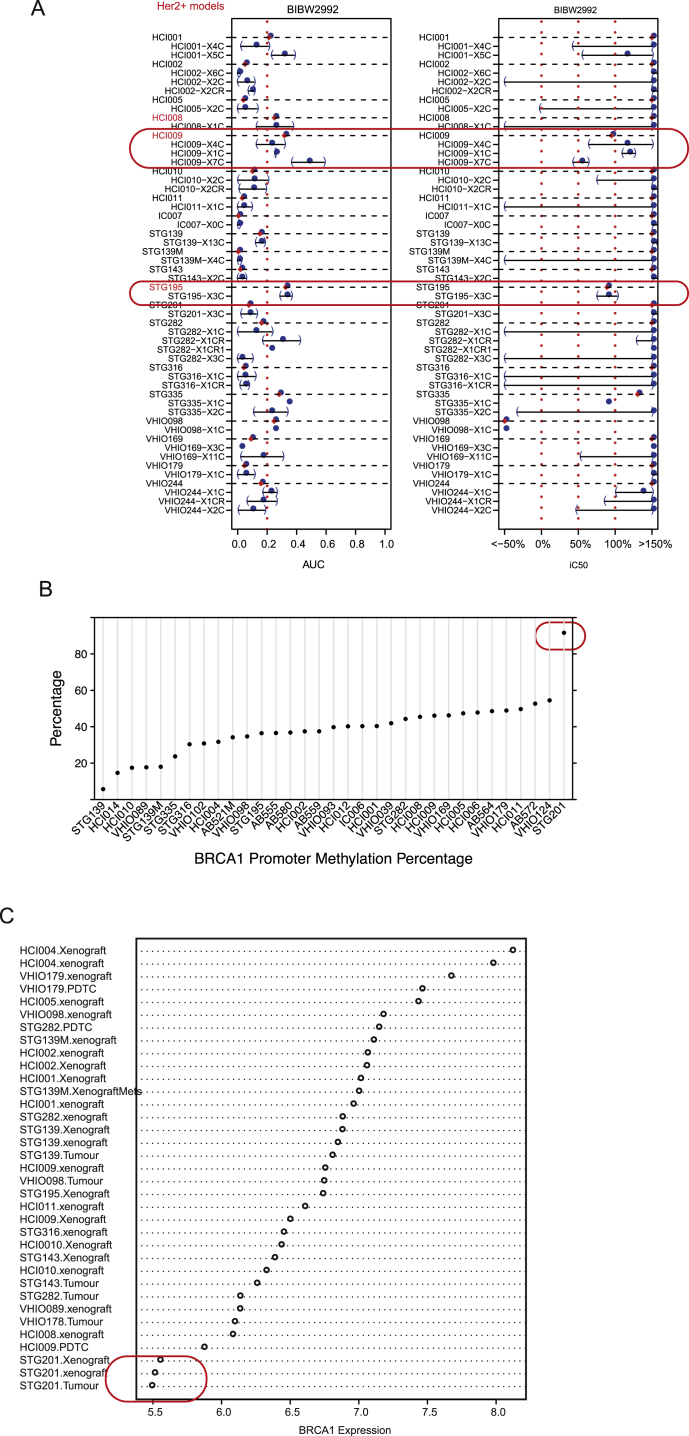
Related to Figure 4 and STAR Methods (A) AUC and iC50 values (as percentage, see STAR Methods) for the EGFR/ERBB2 inhibitor BIBW2992 (Afatinib). Dots represent estimates using all technical replicates and error bars are standard errors of the estimates obtained using each technical replicate individually. (B) BRCA1 promoter methylation percentage measured by RRBS (n = 33 models). (C) BRCA1 expression measured by expression microarrays (n = 35 samples from 19 models).

Third, we explored whether the combined analysis of PDTC drug responses and molecular data recapitulated known mechanisms of drug sensitivity and resistance. For example, sensitivity to the EGFR/ERBB2 inhibitor BIBW2992 (afatinib) was seen in two of the three Her2+ models tested (Figure S6A). Sensitivity to PARP inhibition (Drew et al., 2011) was seen in a model with somatic *BRCA1* promoter methylation and consequent lack of expression (STG201) and in a model from a patient with a germline-truncating *BRCA1* mutation (VHIO124; Figures S6B and S6C; Table S6, and Figure 6 for ex vivo and in vivo data, respectively). Interestingly, two models from *BRCA1* germline mutation carriers were resistant to PARP inhibitors, and these had inactivating mutations of *53BP1* (STG316: c.134+3A > C) and *MAD2L2* (VHIO179: c.66_67delAG; Table S6; Figure 6). Resistance to PARP inhibitors due to loss of non-homologous end-joining (NHEJ) has been previously reported for both *53BP1* (Bouwman et al., 2010, Bunting et al., 2010, Chapman et al., 2012) and *MAD2L2* (Boersma et al., 2015, Xu et al., 2015). These data therefore further demonstrate breast cancer explants recapitulate known mechanisms of both drug sensitivity and resistance.

Finally, we explored multiple layers of molecular data in the context of PI3K pathway inhibition. In Figure 4D, we present a schematic of the PI3K-AKT-mTOR pathway to illustrate the complexity of the associations. Sensitivity to the PI3Kα inhibitor GDC00941 (pictilisib) was seen in models with *PIK3CA*-activating mutations (3/15), *PTEN* loss (5/15), *INPP4B* loss (2/15), high p-AKT levels (4/15), or a combination of these features (Table S6). The difference in response (measured by AUC) to pathway inhibitors was compared in the presence or absence of a biomarker in the pathway (based on expression, SNVs, CNAs, or promoter methylation). This showed models with mutant versus wild-type *PIK3CA* responded better to PI3Kα and AKT inhibitors, but not to mTOR and PI3Kβ inhibitors. We did a similar analysis for JQ1, a BET inhibitor recently tested in breast cancer models (Shu et al., 2016). We tested 19 models, and seven were JQ1 sensitive, including 4/7 ER+ (IntClust1 [3] and IntClust10 [1]) and 3/12 ER− (IntClust10 [2] and IntClust9 [1]; Table S6).

These data highlight the heterogeneous nature of single biomarker/drug-response associations in breast cancer and suggest integrative analysis of molecular and drug response data are more informative. Further improvements are expected in the future using new drug-response metrics that are insensitive to cell division rates.

### Use of PDTCs to Test Drug-Drug Combinations

**Figure 5:**
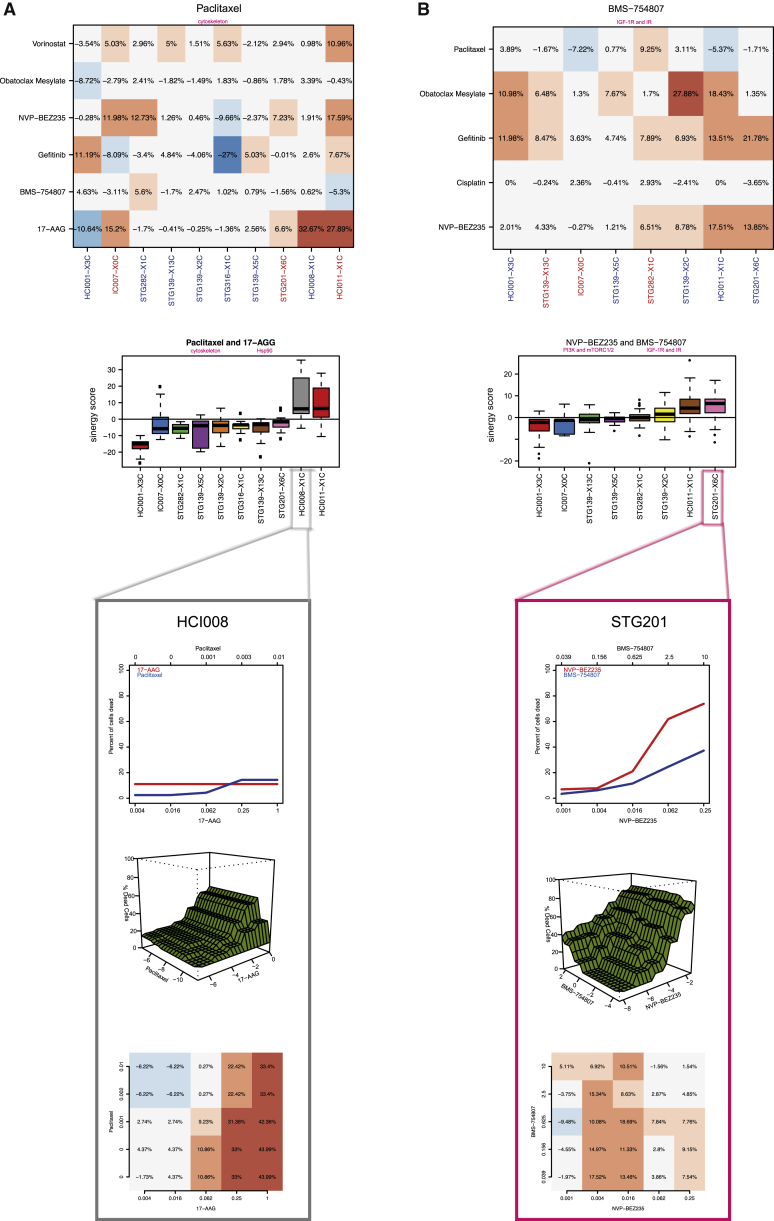
Drug-Drug Combination Studies in PDTCs (A) Synergism of paclitaxel in combination with 17-AAG. (Top panel) Bliss independence model residuals for paclitaxel combinations are shown. The 95% percentile of these differences (in percentage) is plotted. For each drug combination, the expected response is compared to the observed response in all the dose ranges in the combination. (Middle panel) Boxplots of distribution of residuals (Bliss independence model) for paclitaxel and 17-AGG combination in each PDTC model tested are shown. (Bottom panel) Detailed analysis for HCI008 (from top to bottom: single drug curves, bivariate isotonic fit for the combination, and residuals of the Bliss model for each dose combination) is shown. Red shades, synergistic effects; blue shades, antagonistic effects. (B) Synergism of IGF-1R/IR inhibitor (BMS-754807) with PI3K/mTOR inhibitor (NVP-BEZ235). Panels are the same as in A (bottom panel: detailed analysis for STG201).

**Figure S7:**
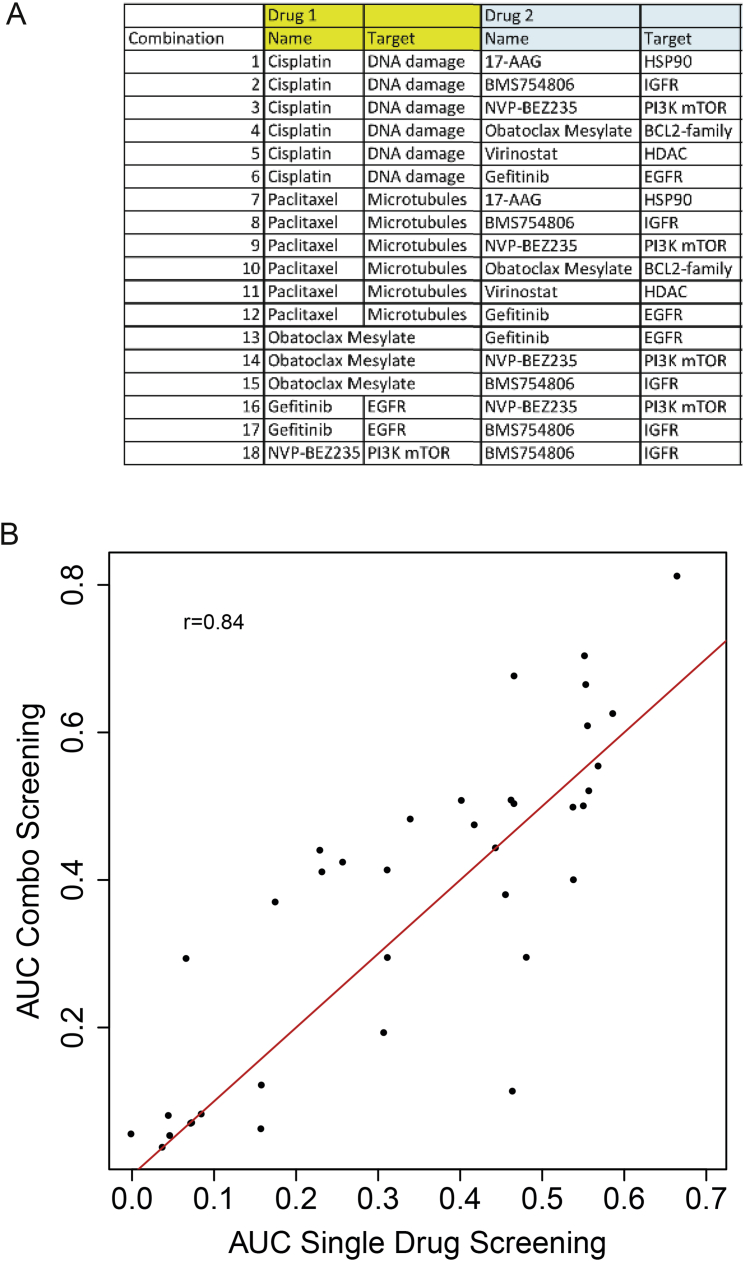
Related to Figure 6 and STAR Methods (A) Table showing drug combinations tested. (B) Scatterplot showing AUC values for all drugs tested in both individual compound screen and as single agent in the drug-drug combination screen. Pearson correlation score is indicated (n = 125 comparisons on 8 models).

Combination therapy is increasingly being used as an approach to combat development of resistance in cancer treatment. To test the use of PDTC models in high-throughput drug-drug combination assays, we designed a 5 × 5 matrix with standard of care chemotherapy agents (cisplatin and paclitaxel) and six clinically relevant targeted compounds (Figure S7A). Single-agent drug responses in these drug-drug combination assays were highly correlated with those obtained from the 108 individual compound screen (Pearson correlation 0.84), further confirming the robust and reproducible performance of our PDTX/PDTC platform (Figure S7B). The Bliss model (see STAR Methods), an approach that does not require precise estimates of IC_50_s, was used to compute synergy and antagonism. The performance of the Bliss model was validated by showing in a Her2-positive model (HCI008) synergy of an Hsp90 inhibitor (17-AAG or tanespimycin) in combination with paclitaxel, which has been previously reported (Modi et al., 2011; Figure 5A). The testing of pairwise combinations using the six targeted compounds (Figure S7A) confirmed the rationally predicted synergistic effects of combining an IGFR1/INSR1 inhibitor (BMS-754807) with a dual PI3K/mTOR inhibitor (NVP-BEZ235) or an EGFR inhibitor (gefitinib/Iressa; Figure 5B).

In summary, these data show that PDTCs can be successfully used to test drug-drug combinations.

### PDTC Testing Predicts In Vivo Drug Responses in PDTXs

**Figure 6:**
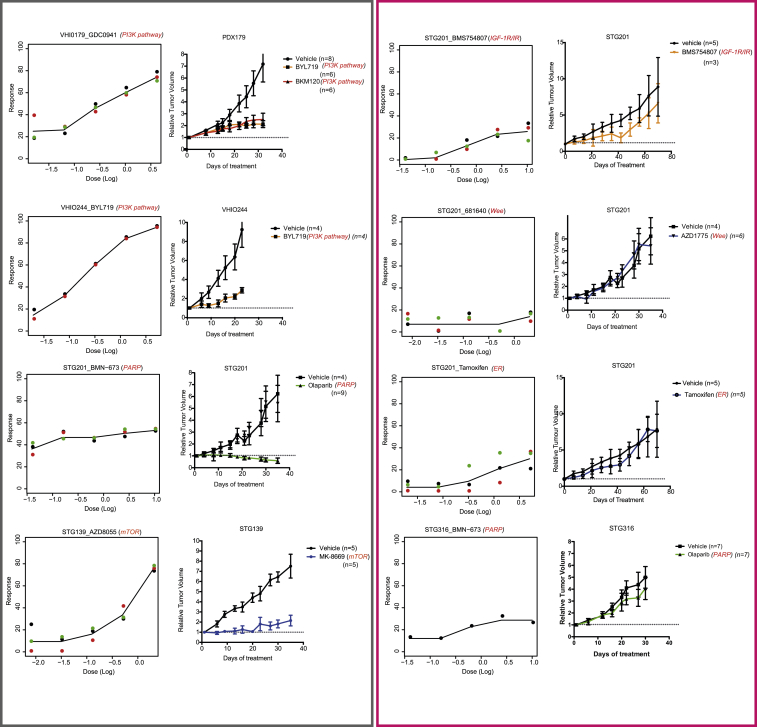
Validation of Ex Vivo PDTC Drug Responses with In Vivo PDTX Testing Representative sensitive (gray panel) and resistant (pink panel) drug responses in several models. (Left plots) PDTC ex vivo dose response is shown. (Right plots) PDTX in vivo tumor growth curves are shown (sample sizes are indicated in the plot; average values and error bars representing SDs are shown). See also Figure S7.

We next tested whether PDTC drug responses ex vivo predict responses in vivo in a series of pre-clinical trials using PDTXs as xenopatients. This step was crucial to validate the utility of the platform reported here, given that PDTXs have recently been shown to predict human clinical trial drug responses (Gao et al., 2015). We selected 40 ex vivo PDTC drug responses tested in eight different models for in vivo validation. Significantly, even though different compounds with the same specificity had sometimes to be used in PDTXs (for formulation or bioavailability reasons), 33 out of 40 (82.5%) ex vivo drug responses were recapitulated in vivo (Figure 6 for examples; Table S7 for details on all ex vivo and in vivo drug tests performed). This included validation of responses in vivo for PI3K-AKT-mTOR pathway, ER, PARP, Wee1, and IGFR1 inhibitors (Figure 6A; Table S7). We also validated in vivo the predicted synergistic combinations of PI3K plus IGFR1/INSR1 inhibitors (Table S7).

Overall, these results show the value of PDTCs as predictive drug response models prior to in vivo testing using PDTXs.

## Discussion

The use of PDTXs in pre-clinical cancer drug development has become widespread (Crystal et al., 2014, Marangoni and Poupon, 2014, Messersmith et al., 2009). The data available (DeRose et al., 2011, Eirew et al., 2015, Messersmith et al., 2009), to which we add extensively here, show PDTXs share most molecular and architectural features with their originating patient tumor sample. A recently published large study of 1,000 PDTX models adds a further crucial piece of evidence supporting their potential utility by showing the use of xenograft models to predict human clinical trial drug responses (Gao et al., 2015). The dataset presented here shows the unique value of a living biobank of breast cancer explants that preserve intra-tumor heterogeneity as a platform for drug screening, including the demonstration of reproducible drug responses across different xenograft passages.

A significant limitation of PDTXs as a pre-clinical platform is the fact that in vivo studies are not well suited for high-throughput drug screening. The PDTX/PDTC platform presented here overcomes this limitation, and we have demonstrated its use for both high-throughput single and drug-drug combination studies. The platform has remarkably good reproducibility and selectivity, similar to that observed in analogous studies using cell lines or organoids (Garnett et al., 2012, van de Wetering et al., 2015). The demonstration that compounds affecting the same pathway or target and those with similar mechanisms of action shared the same drug responses across models testifies to its biological robustness. We independently tested a set of drug responses in a selection of models with a DNA-based method, showing very good correlation with CTG results (which is based on ATP levels), as others have recently reported (Haverty et al., 2016). Further refinement of the in vitro screening will come from introducing growth rate inhibition metrics (Hafner et al., 2016). The in vivo validation of 33 out of 40 in-vitro-predicted drug responses tested suggests that, in the future, PDTCs can be used as a drug-screening platform prior to downstream testing with the 1X1X1 PDTX clinical trial design (Gao et al., 2015).

Crucially, we found that PDTXs and PDTCs are communities of clones of varying complexity and that these explants display intra-tumor heterogeneity similar to that that is found in the clinical population. The preservation of clonal communities within heterogeneous tumors in pre-clinical models has recently re-emerged as key to improving therapeutic strategies (Heppner, 1984, Tabassum and Polyak, 2015). This feature uniquely positions PDTXs as a human pre-clinical model to study breast cancer biology and drug responses.

The framework we developed of ex vivo PDTC drug screening followed by in vivo PDTX response validation is a cost-effective pipeline for pre-clinical drug development. The extensive detailed STAR Methods accompanying this report, including both processed and raw molecular profiling and drug sensitivity information, constitutes a publicly available dataset that we will continue to expand with more models and further drug testing. We will provide viable xenograft fragments to academic collaborators and will also make models available to the wider community through licensing. The extensive data generated already represent a valuable resource, which can be easily browsed in a purpose-built public web portal (http://caldaslab.cruk.cam.ac.uk/bcape). We are using the PDTX/PDTC platform to study mechanisms of drug resistance, to unravel clonal dynamics in response to therapeutic perturbation, and to perform genome-wide perturbations with small hairpin RNA (shRNA) and CRISPR-CAS libraries (Marcotte et al., 2016, Shalem et al., 2015), and these newly generated data will be continually deposited into the public domain.

## STAR★Methods

### Key Resources Table

**Table:** 

REAGENT or RESOURCE	SOURCE	IDENTIFIER
**Antibodies**		

Mouse monoclonal (LL002) CK14	Novocastra	Cat # LL002; RRID: AB_892359
Rabbit monoclonal (E431-1) anti-Cytokeratin 18	Abcam	Cat # ab32118; RRID: AB_736394
Mouse monoclonal (ER 6F11) anti-ER	Novocastra	Cat # NCL-ER-6F11/2
Mouse monoclonal (PgR 636) anti-PR	Dako	Cat # M3569; RRID: AB_2532076
Mouse monoclonal (XM26) anti-CK5	Novocastra	Cat # NCL-CK5
Mouse monoclonal (TS1) anti-CK8	Novocastra	Cat # NCL-CK8
Mouse monoclonal (NCH-38) anti-E-Cadherin	Dako	Cat # M3612; RRID: AB_2076672
Mouse monoclonal (VU-1D9) anti-ESA	Novocastra	Cat # NCL-ESA
Rabbit monoclonal (91B2) anti-Phospho-S6 Ribosomal Protein (Ser235/236)	Cell Signaling Technology	Cat # 4857
Mouse monoclonal (MIB-1) anti-Ki67	Dako	Cat # M7240; RRID: AB_2142367
Mouse monoclonal (D0-7) anti-P53	Dako	Cat # M7001
Rabbit Monoclonal (4B5) anti-HER-2/neu	Roche	Cat # 790-2991
FITC anti-mouse H-2K^b^/H-2D^b^ (28-8-6)	Biolegend	Cat # 14605;

**Chemicals, Peptides, and Recombinant Proteins**		

Matrigel	Corning	Cat #354230
DMEM/F12/HEPES	GIBCO	Cat # 11330-057
Collagenase	Roche	Cat #11088793001
Hyaluronidase	Sigma	Cat #H3506
BSA fraction V	GIBCO	Cat #15260-037
Insulin	Sigma	Cat #I6634
Gentamycin	GIBCO	Cat #15750-060
Dispase	StemCell technologies	Cat #7913
DNase	Sigma	Cat #D4513
Ammonium Chloride	StemCell technologies	Cat #7850
Trypsin	GIBCO	Cat #7400
MEGM	Lonza	Cat # CC-3150
DMSO	Sigma	Cat #D8418

**Critical Commercial Assays**		

CyQUANT Direct Cell Proliferation Assay	Thermo Fisher Scientific	Cat # C35011
CellTiter-Glo Luminescent Cell Viability Assay	Promega	Cat # G7570
SYTOX Green Nucleic Acid Stain	Thermo Fisher Scientific	Cat # S7020
PKH26	Sigma	Cat# Mini26-1KT

**Deposited Data**		

Raw sequencing/raw microarray data	EGA	EGAS00001001913
Normalized data files	figshare	https://figshare.com/s/4a3f6bc543e5ba85834c

**Experimental Models: Organisms/Strains**		

NSG mice	Charles River	NOD.Cg-Prkdcscid Il2rgtm1Wjl/SzJ

**Sequence-Based Reagents**		

Star^∗^FISH© Ready-to-use Human Chromosome Pan-Centromeric paints	Cambio	1695-F-01
Star^∗^FISH Ready-to-use Mouse Chromosome Pan-Centromeric paints	Cambio	1697-MF-01

**Software and Algorithms**		

annovar March 2015	Wang et al., 2010	http://annovar.openbioinformatics.org/en/latest/
ASCAT 2.2	Van Loo et al., 2010	https://www.crick.ac.uk/peter-van-loo/software/ASCAT
Bioconductor 3.2	Huber et al. (2015)	http://www.bioconductor.org
Bioconductor package beadarray 2.18.0	Dunning et al., 2007	http://www.bioconductor.org
Bioconductor package CopywriteR 2.0.6	Kuilman et al., 2015	http://www.bioconductor.org
Bioconductor package DNACopy 1.46.0	Olshen et al., 2004	http://www.bioconductor.org
Bioconductor package genefu 1.1.8.0	Haibe-Kains et al., 2012	http://www.bioconductor.org
Bioconductor package gsva 1.20.0	Hänzelmann et al., 2013	http://www.bioconductor.org
Bioconductor package QDNaseq 1.2.4	Scheinin et al., 2014	http://www.bioconductor.org
Bioconductor package VariantAnnotation 1.12.9	Obenchain et al., 2014	http://www.bioconductor.org
Bismark 0.14.0	Krueger and Andrews, 2011	http://www.bioinformatics.babraham.ac.uk/projects/bismark/
bwa 0.7.9	Li and Durbin, 2009	http://bio-bwa.sourceforge.net/
GATK 3.3.0	DePristo et al., 2011	https://software.broadinstitute.org/gatk/
In-house algorithms for pam50	Originally in Parker et al., 2009. The version used here is described in Curtis et al., 2012.	http://www.nature.com/nature/journal/v486/n7403/full/nature10983.html
In-house algorithm for Three-Gene classification	Originally in Haibe-Kains et al., 2012. The version used here is based on the genefu package implementation and is described in Ali et al., 2014	https://static-content.springer.com/esm/art%3A10.1186%2Fs13059-014-0431-1/MediaObjects/13059_2014_431_MOESM17_ESM.zip
Novoalign 3.2	Novocraft	http://www.novocraft.com/products/novoalign/
Picard tools 1.85	Picard	https://broadinstitute.github.io/picard/
PyClone 0.12.7	Roth et al., 2014	http://compbio.bccrc.ca/software/pyclone/
R 3.2.0	R Core Team, 2016	http://www.r-project.org
R package deconstructSigs 1.6.0	Rosenthal et al., 2016	http://www.cran.r-project.org
R package flux 0.3.0.	Jurasinski et al., 2014	http://www.cran.r-project.org
R package iC10 1.2.0	Ali et al., 2014	http://www.cran.r-project.org
R package isotonic.pen 1.0	Meyer et al., 2014	http://www.cran.r-project.org
R package mclust 5.2	Fraley and Raftery, 2002	http://www.cran.r-project.org
R package mgcv 1.8.12	Wood, 2004	http://www.cran.r-project.org
samtools 1.2	Li et al., 2009	http://www.htslib.org/

**Other**		

1000 genomes database Oct 2014	Abecasis et al., 2012	http://www.1000genomes.org
C6 oncogenic signatures database	Subramanian et al., 2005, Tamayo et al., 2011.	http://software.broadinstitute.org/gsea/msigdb/collections.jsp#C6
dbSNP database 138	Sherry et al., 2001	http://www.ncbi.nlm.nih.gov/projects/SNP/
GenomicSuperDups database libj26	Bailey et al., 2002	http://varianttools.sourceforge.net/Annotation/GenomicSuperDups
MetaLR database libj26	Dong et al., 2015	https://sites.google.com/site/jpopgen/dbNSFP
Mutation Taster database libj26	Schwarz et al., 2010	http://www.mutationtaster.org
Polyphen 2 database libj26	Adzhubei et al., 2013	http://genetics.bwh.harvard.edu/pph2/
SIFT database libj26	Kumar et al., 2009	http://sift.jcvi.org
TCGA breast cancer mutation data	Cancer Genome Atlas Network, 2012	http://www.nature.com/nature/journal/v490/n7418/full/nature11412.html
Female Silhouette icon	Human body diagrams	https://commons.wikimedia.org/wiki/Human_body_diagrams
Mouse icon	Open Clipart	https://openclipart.org/

### Contact for Reagent and Resource Sharing

Further information and requests for material may be directed, and will be fulfilled by the corresponding author Carlos Caldas (carlos.caldas@cruk.cam.ac.uk).

### Experimental Model and Subject Details

#### Generation and Maintenance of a Living Biobank of Human Breast Cancer Explants

A bank of human breast cancer explants has been maintained at the CRUK Cambridge Institute (Figure 1A and Table S1) over the past 4 years, by combining efforts from Addenbrookes Hospital and collaborating hospitals in Europe (Institute Curie, Paris and VHIO, Barcelona), US (Huntsman Cancer Institute, Salt Lake City, Utah) and Canada (UBC, Vancouver). The PDTX biobank in Cambridge continues to expand with routine implantation of, on average, 3 breast cancer samples per week. The time from patient collection to mouse implantation ranges from 30-180 min.

Surgically resected primary breast cancer tissue, biopsies from brain, skin, liver, bone, axilla and lymph node metastasis, and pleural effusions or ascites samples were obtained from consenting patients. The research was done with the appropriate approval by the National Research Ethics Service, Cambridgeshire 2 REC (REC reference number: 08/H0308/178). Tissue samples were embedded in matrigel and then implanted subcutaneously into 2-4 female severe immune compromised NSG mice. Pleural effusion and ascites samples were centrifuged, washed with water twice to eliminate red blood cells, and cell pellets resuspended in 50% matrigel:FBS solution before subcutaneous injection into mice.

PDTXs were serially implanted into multiple hosts to allow in vivo expansion of each model (established model). Xenograft samples were cryopreserved in liquid nitrogen and freezing media (FBS/10%DMSO) at each passage, from each mouse. Genotyping of all samples was always performed to confirm matching with the originating patient derived sample (see below). All models tested to date could be rescued by re-implantation of cryopreserved tissue. This includes successful implantation of PDTX samples obtained from the network of collaborators, which were all obtained under the appropriate Institutional Review Boards and transferred to Cambridge under Materials Transfer Agreements. All animal experiments were conducted in compliance with the rigorous Home Office framework of regulations (Project License 707679).

#### Generation of Viable PDTX-Derived Tumor Cells

Xenograft tissue, either freshly collected or cryopreserved in FBS/10%DMSO was minced using sterile scalpels and dissociated for a maximum of 90 min in DMEM/F12/HEPES (GIBCO), 1mg/ml Collagenase (Roche), 100U/ml Hyaluronidase (Sigma), 25% BSA fraction V (GIBCO), 5 μg/ml Insulin and 50 μg/ml Gentamycin (GIBCO). This was followed by further dissociation using trypsin (GIBCO), Dispase (StemCell technologies) and DNase (Sigma). Red blood cell lysis was done by washing the cell pellet in a 1:4 solution of HF media (GIBCO): Ammonium Chloride (StemCell technologies). Cells were resuspended in MEGM (Lonza) and filtered through a 40 μm filter. For high throughput drug screens, cells were plated in MEGM in 384 well plates at a concentration of 1x10^6^ cells/ml. Other in vitro assays used cell concentrations outlined in the individual methods.

#### Sample Nomenclature

Each sample ID follows the structure XXXX-A0C0, where

XXXX: name of the model

A: Type of sample (T: tumor, N: normal, X: xenograft)

0: If the sample is a xenograft, number of passage (starting with zero)

C: C indicates PDTC

0: Number of days of culture

Any R, R1 in the name indicates a replicate.

#### Sample labeling

In Eirew et al. (2015), STG139 was labeled as SA577, STG143 as SA536 and STG201 as SA541.

### Method Details

#### Histopathological Review

Tissue microarrays were prepared using duplicate 0.6mm cores extracted from formalin-fixed paraffin-embedded blocks containing material from patient tumors and xenografts.

These were run using Leica’s Polymer Refine Kit on their automated Bond platform. The HIER’s (sodium citrate and tris EDTA pre-treatments) are all run at 100°C, for the time indicated in the table. The DAB Enhancer (used for all antibodies apart from ER and PR) reference is AR9432. The de-waxing and re-hydration prior to IHC are done on the automated Leica ST5020, as is the post-IHC de-hydration and clearing. Finally, the mounting is done on Leica’s CV5030. The slides were reviewed by a pathologist.

#### PKH26 Assay

PKH26 assay (Sigma) was performed as per manufacturer’s instructions. Briefly; 1x10^7^ cells from xenograft single cell suspensions were incubated for 5 min in 2x10^−6^M PKH26. Cells were then cultured in suspension in MEGM media (Lonza). Aliquots were taken periodically, and fixed in 2% Neutral buffered formalin prior to flow cytometric analysis.

#### Centrosome FISH

Pan-centromeric FISH was performed using human and mouse specific Star^∗^FISH© chromosome paints (Cambio). The protocol was performed on 3-6 micron FFPE tissue sections as per manufacturer’s instructions.

#### Flow Cytometric Analysis of Xenograft Mouse Stromal Cell Content

Frozen xenograft samples were prepared as single cell suspensions. Non-specific antibody labeling was blocked by incubation in 10% normal rat serum for 30 min. Percentage mouse stromal content was quantified using FITC anti-mouse H-2Kb/H-2Db Antibody [Clone: 28-8-6] (Biolegend UK Ltd).

#### Cell Viability Assays

Single cell suspensions generated from a 1.5 cm^3^ PDTX tumor were plated in triplicates at 40.000 cells/well into 384-well plates. Drug was added to wells after 24h. To quantify drug responses in PDTCs, cell viability reading intensities were obtained 6 days post-treatment and normalized against positive and negative values. To independently validate data from our drug screening approach using CellTiter-Glo (CTG), a selection of 8 models were screened with 18 drugs and cell viability determined in parallel by CTG and two further tests (CyQUANT and SYTOX).

1) CellTiter-Glo (CTG): The methodology was adapted from the protocol previously described for cell lines (Garnett et al., 2012).
2) CyQUANT Direct Cell Proliferation Assay (C35011). The manufacturer’s instructions were adapted to suit PDTC culture. Briefly, 20 μl of 10X detection reagent was added to 70 μl of drug treated cells. The cells were incubated at 37°C and Fluorescence was read on the Pherastar plate reader after both 1 and 8 hr of incubation.
3) SYTOX Green Nucleic Acid Stain - 5 mM Solution in DMSO (S7020). Briefly, 500mM EDTA (pH 7.0) stock diluted 1:100 in TBS, filtered through 0.45 μm membrane was used as dilution buffer. 5mM SYTOX stock was diluted 1:1000 in dilution buffer and 10 μl was added to cells. The cells were incubated at room temperature for 6 hr. 5 μl of 1% Saponin solution with 0.04% Sodium Azide was added to the cells and incubated for 20 hr at room temperature. Fluorescence was read on the Pherastar plate reader. These comparisons were performed using 10 doses per drug.

#### Treatment of PDTXs In Vivo

The following reagents were used for in vivo validations upon randomization of tumor bearing NSG mice: MK-8669/Ridaforolimus as an allosteric mTORC1 inhibitor (1mg/kg, 5IW), BKM120/Buparlisib as a pan-PI3K and BYL719 and GDC0032/Taselisib as PI3K-alpha inhibitors (27.5, 35mg/kg 6IW and 5 mg/kg, respectively), LEE011/Ribociclib as a CDK4/6 inhibitor (75mg/kg), AZD2281 (Olaparib/Lynparza) as a PARP inhibitor (50mg/kg, 5IW), AZD1775 as Wee1 inhibitor (120mg/kg, 5dON 9dOFF) and Tamoxifen (10mg/ml, 100μl daily) as an ER pathway inhibitor. Tumor volumes were normalized to the starting tumor volume and mean volumes were plotted and compared to vehicle-treated controls. Details such as number of mice used and mean volumes per treatment arm are shown in Table S7.

All experimental procedures were approved by the University of Cambridge Animal Welfare and Ethical Review Committee and by the Vall d’Hebron Hospital Clinical Investigation Ethical Committee and Animal Use Committee.

#### Experimental Design

One of the main goals of this project was to study variability in the molecular features of the tumors engrafted and the drug responses in PDTCs. For that, different levels of replication were used: tumor biological replicates (where different pieces of originating cancer sample were engrafted), PDTX biological replicates (where different mice were engrafted with different samples from the same PDTX sample), PDTC biological replicates (where the same PDTX was cultured at least twice as PDTCs) and technical replicates for both PDTXs and PDTCs.

No specific strategy for randomization was employed, and no blinding was used, except for in vivo validations of drug response as described in the previous section. As this is a pilot study and we had no prior estimates of variability between engraftments, no sample size calculations were done. There were no criteria of exclusion for tumor engraftment.

### Quantification and Statistical Analysis

Throughout all the analysis description, we refer to “model” as a collection of a primary tumor, matched normal samples, matched PDTX passages or PDTCs, and “sample” as any of the instances of a given model.

Statistical parameters including the exact value of n in terms of number of samples and models and the definition of location and dispersion measures for each figure are reported in the Figures and the Figure Legends.

#### Details and Number of Samples Analyzed

##### Whole-Genome Sequencing

Number of samples sequenced: 1

Number of different models: 1

STG139M

##### Whole-Exome Sequencing

Number of samples sequenced: 193

Number of different models: 33

AB521M, AB551, AB555, AB559, AB580, AB630, CAMBMT1, HCI001, HCI002, HCI004, HCI005, HCI008, HCI009, HCI010, HCI011, IC007, STG139, STG139M, STG143, STG195, STG201, STG282, STG316, STG335, VHIO039, VHIO089, VHIO093, VHIO098, VHIO102, VHIO124, VHIO131IGFRES, VHIO179, VHIO244

##### Shallow Whole-Genome Sequencing

Number of different samples: 132

Number of different models: 32

AB521M, AB551, AB555, AB559, AB580, AB630, CAMBMT1, HCI001, HCI002, HCI004, HCI005, HCI008, HCI009, HCI010, HCI011, STG139, STG139M, STG143, STG195, STG201, STG282, STG316, STG335, VHIO039, VHIO089, VHIO093, VHIO098, VHIO102, VHIO124, VHIO131IGFRES, VHIO179, VHIO244

##### Reduced Representation Bisulfite Sequencing

Number of samples sequenced: 68

Number of different models: 33

Average number of CpGs with at least 5 reads: 2,355,537

AB521M, AB555, AB559, AB564, AB572, AB580, HCI001, HCI002, HCI004, HCI005, HCI006, HCI008, HCI009, HCI010, HCI011, HCI012, HCI014, IC006, STG139, STG139M, STG195, STG201, STG282, STG316, STG335, VHIO039, VHIO089, VHIO093, VHIO098, VHIO102, VHIO124, VHIO169, VHIO179

##### Expression Arrays

Number of samples: 153

Number of different models: 39

AB521M, AB551, AB555, AB559, AB580, AB630, HCI001, HCI002, HCI004, HCI005, HCI006, HCI008, HCI009, HCI010, HCI011, HCI014, IC006, IC007, STG139, STG139M, STG143, STG195, STG201, STG282, STG316, STG335, VHIO006, VHIO039, VHIO089, VHIO093, VHIO094, VHIO098, VHIO102, VHIO124, VHIO131, VHIO161, VHIO169, VHIO179, VHIO244

##### Drug Screening

Single-Drug Tests:

Number of total single-drug tests (with technical replicates): 6634

Number of single-drug tests: 2550

Number of different models: 20

Number of different models/passages: 37

Number of different drugs: 104

Combinations:

Number of total combination tests (with technical replicates): 288

Number of combination tests: 144

Number of different models: 8

Number of different models/passages: 10

Number of different combinations: 18

HCI002, HCI009, HCI010, HCI001, HCI005, HCI008, HCI011, IC007, STG139, STG139M, STG143, STG201, STG195, STG282, STG316, STG335, VHIO098, VHIO179, VHIO169, VHIO244

### Computational Pipeline for Discriminating Mouse and Human Sequences

The analysis of sequencing data from PDTXs is hampered by the presence of mouse stroma contamination. Due to the high homology between the two genomes, a proportion of mouse reads can still map to homologous regions of the human genome, likely with some mismatch, heavily affecting downstream analysis.

To develop a computational approach able to tackle this issue, we carried out a Whole Exome Sequencing (WES) experiment in a controlled setting where fixed amounts of human and mouse DNA were mixed, ranging from a pure human sample to a pure mouse sample.

The adopted strategy was to align the reads against a combined human-mouse genome with Novoalign. This way, although mouse reads could be mapped against the human genome, they will likely map with higher score in the corresponding mouse locus. Reads mapping with identical scores in two (or more) locations were discarded.

The table below shows the performance of our approach. When a pure human DNA sample or a pure mouse DNA sample is mapped against the combined genome, 99.9% of the reads or more are mapped correctly.

**Table:** 

Sample ID	% human DNA	Replicate	% reads mapped on human genome	% reads mapped on mouse genome	Estimated human DNA content
0_100_R1	0	1	0.10	99.90	0.00
0_100_R2	0	2	0.10	99.90	0.01
0_100_R3	0	3	0.08	99.92	0.01
25_75_R1	25	1	62.44	37.56	22.55
25_75_R2	25	2	64.58	35.42	24.36
50_50_R1	50	1	83.14	16.86	52.59
50_50_R2	50	2	82.18	17.82	50.56
50_50_R3	50	3	82.78	17.22	51.82
90_10_R2	90	1	97.59	2.41	90.97
90_10_R3	90	2	97.58	2.42	90.93
100_0_R1	100	1	99.98	0.02	98.70
100_0_R2	100	2	99.98	0.02	98.69
100_0_R3	100	3	99.98	0.02	98.70

We found a non-linear relationship between the percentage of human DNA content and the percentage of reads mapped on the human genome. The bias is likely caused by the capturing step, since probes specifically designed against human exons were used. Therefore, we derived a calibration curve using a loess regression (Figure S2B), making it possible to estimate the human DNA content in independent samples.

### WES Analysis

WES libraries were prepared using Nextera Rapid Capture Exome (Illumina Inc., USA) following manufacturer’s instructions (Enrichment Guide version #15037436 Rev. J, Illumina Inc., USA). Briefly, PDTX DNA was quantified using Quant-iT broad range dsDNA Assay (Thermo Fisher, USA) and 50ng used as input into the Nextera exome library preparation. Pre-capture libraries were normalized to 20nM and pooled for shallow whole genome sequencing (sWGS). Pre-capture libraries were quantified using qPCR and their average length was assed using DNA1000 chip on Bioanalyzer 2100 (Agilent Technologies Inc., USA). 500ng of each library was pooled for three-plex exome capture, and after 11 cycles of PCR amplification were again normalized to 15nM and pooled for high-coverage paired-end exome sequencing.

The sequencing was performed using 125bp paired-end reads. Short reads were aligned using novoalign (Novocraft) with our custom pipeline to remove mouse contamination. Bam files were merged, sorted and indexed using samtools. Duplicates were marked using Picard tools and insertions and deletions (indels) were realigned using GATK.

For quality control purposes and to check for sample labeling mistakes, all samples were genotyped using GATK HaplotypeCaller and a few errors were identified and corrected. HaplotypeCaller was also employed for variant calling, and after that several filters were applied using the Bioconductor package VariantAnnotation: for single nucleotide variants (SNVs), a minimum genotyping quality of 20, at least 5 reads at the variant position, a strand bias Phred-scale p value smaller than 40 and no presence of homopolymers in the surrounding region. For indels, we increased the width of the region to detect nearby homopolymers. Genotypes and variant allele frequencies (VAFs) were computed from these calls.

All variants were annotated using annovar version March 2015 for gene/exon annotation, 1000 genomes version Oct 2014, dbSNP version138, repetitive regions genomicSuperDups database, SIFT, Polyphen 2, MutationTaster and MetaLR, all versions ljb26.

Variants in intergenic, intronic or ncRNA intronic positions were discarded.

In order to quantify variability in matched tumor/PDTX, different passages of PDTXs or matched PDTX/PDTC, all variants detected in at least one sample of each model were obtained. For those samples where those variants had not been detected GATK HaplotypeCaller was run again on those positions to see if this was a consequence of no reads in that region for that sample or a real absence of the variant.

Normal contamination estimates were obtained for each tumor sample combining copy number calls from shallow sequencing (see next section) and SNV calls. First, we selected heterozygous SNPs in the matched normal sample (if available, otherwise all heterozygous variants in the tumor were considered), and then looked at only those in regions of copy number loss in the tumor, as defined by a segmented mean copy number log ratio smaller than −0.1. In these regions, as a loss of heterozygosity has occurred, it is expected that all variants will have a VAF of 0 or 1 (if no normal contamination is present). For the AB SNPs that are left to B genotype, the expected value of contamination would be 2 ^∗^ VAF – 1, assuming that all tumor cells acquired the deletion. As this is a downward estimate, we chose as the tumor content estimate the maximum of the density function of the VAF of those variants. These estimates were used to correct tumor VAFs or copy numbers where needed in the rest of the analyses.

All variants that were present in the 1000 Genomes database or in any of our normal samples were labeled as germline. Regions marked as repetitive were also filtered, and insertions that represented a segmental duplication were removed if they were not present in at least three-fourths of all the samples for a given model or in 3 of them. Somatic variants that were not filtered were compiled for each model. Some manual curation was needed for genes like PI3KCA, where variants from a region of segmental duplication were included after manual inspection.

Frequencies of mutations were compared to TCGA.

Pearson correlations between VAFs in tumor and PDTX, different passages of the same model, PDTX and PDTCs of the same passage, mice replicates, technical replicates of the same sample and different tumors were computed.

To quantify tumor heterogeneity two methods were used: MATH (Mroz and Rocco, 2013) and PyClone (Roth et al., 2014). MATH essentially quantifies the ratio of the width of the center of the VAF values, while PyClone is a Bayesian clustering method that infers the clonal population structures for each sample from each model. Briefly, PyClone takes as input the allele frequencies of somatic mutations in each sample and clusters mutations that shift together across the samples, predicting the cellular frequency for each cluster in each sample accounting for copy number changes and normal cell contamination. Because PyClone requires absolute allelic copy number information to be inputted for each mutation, ASCAT v2.2 was ran for that task. ASCAT was run using the log ratios from the shallow sequencing when available and the VAFs from the exome sequencing. For those samples with no shallow sequencing, CopywriteR was run on the whole exome sample. When possible, tumor/normal paired analysis was run on ASCAT. Filtering and quality control was carried out prior to running ASCAT; samples were removed if the mean coverage across all the mutations was less than 20. Mutations that were not sequenced to a depth of at least 10 reads in either the cancer or normal sample were also excluded.

We then applied Pyclone to a total of 22 PDTX models, ranging between 2 and 15 samples for each model. Only mutations with a median coverage of at least 50 across all model samples were used. Additionally, for each model, mutations with a coverage smaller than 15 in any of the samples were removed from the analysis. Germline mutations (SNPs, duplications, and repeats present in the normal sample) were not utilized (but note that germline filtering was less stringent for PyClone analysis; in particular, variants that were present in the 1000 Genomes database were not removed, as we observed some VAF changes between samples that would be useful to distinguish variability from real changes). Mutations with low variant allele frequency (< 0.1) across all samples were also excluded. In cases where greater than 300 high quality mutations remained after filtering, the 300 mutations with the highest median coverage were used as input for PyClone. Normal cell contamination estimations for each tumor sample were also used (the PDTX and PDTC samples have a theoretical contamination of 0). PyClone was run for 40,000 iterations with a burn in period of 20,000 iterations using a beta binomial parameter of 500. To measure the clones whose prevalence changed significantly between time points, we compute the 90% credible intervals and those clusters that had a sample not overlapping with the rest were called significant. These mutations were considered cancer driver genes if they were part of the 40 MutDriver genes (Pereira et al., 2016). For clarification, and as recommended in the original PyClone paper, we only plotted those clusters with more than one mutation.

Mutational profiles (Alexandrov et al., 2013) were computed for each of the samples using the package deconstructSigs (Rosenthal et al., 2016). Signatures were not computed due to the small number of variants in many samples.

### sWGS

The sWGS workflow uses low-coverage sequencing of the pre-capture exome-sequencing libraries to generate high-quality CNA calls. This is efficient in PDTX DNA usage, low-cost (around £30 per sample), and generates better quality copy-number data than what is achievable from WES. As described above in exome sequencing analysis, libraries were prepared using Nextera Rapid Capture Exome (Illumina Inc., USA). Pre-capture libraries were normalized to 10uM and pooled for shallow whole genome sequencing (sWGS).

50 bp single-read whole-genome shallow sequencing was performed in parallel with the exome sequencing to provide a clean and accurate estimate of copy number. Alignment was performed using bwa with our custom pipeline to remove mouse contamination. Bam files were merged, sorted and indexed using samtools. Duplicates were marked using Picard tools. The data were analyzed using the Bioconductor package QDNaseq. This method divides the genome in regions of 100Kb and counts all the reads within those bins. Those reads are then corrected for mappability and GC content and segmented using DNAcopy. Some additional filtering was applied to account for regions not properly mapped.

We also observed that in a few occasions, the method shifted the log2 ratios because of the median normalization. Those cases were inspected perusing the VAF plots obtained with ASCAT and fixed.

The segmented means of the tumors were corrected for normal contamination (as described in the exome pipeline) and copy numbers (HOMD, Homozygous deletions, HETD, Heterozygous deletions, NEUT, neutral copy number, GAIN, single copy gains and AMP, high-level amplifications), were called based on thresholds on the segmented mean log2-ratio (−1, −0.4, 0.25, 0.75).

Pearson correlations between segmented means in tumor and PDTX, different passages of the same model, PDTX and PDTCs of the same passage, mouse replicates, technical replicates of the same sample and different tumors were computed.

### Microarray Expression Analysis

RNA expression was analyzed using the Illumina HT-12 v3 platform. Raw data were processed with the beadarray package. The BASH algorithm was employed to correct for spatial artifacts. Summarization and probe selection based on quality was performed on the bead-level data using the detection thresholds recommended in the package and the re-annotation of the Illumina HT-12v3 platform as described previously (Curtis et al., 2012). The samples were classified into the intrinsic subtypes using PAM50, and the Three Gene classifier. ER, Her2 and PR status was inferred fitting a mixture model with the package mclust.

Classification into Integrative Clusters (IntClusts). The PDTX samples were classified into one of the 10 Integrative Clusters (Curtis et al., 2012) using the scripts provided in (Ali et al., 2014) and the R package iC10 (Ali et al., 2014). Each sample was classified into the 10 Integrative Clusters and the assignment to each model was done by consensus (that is, the majority group for all the samples from that model). Copy number and expression data were used when available (n = 21). If not, only expression data (n = 17) or only copy number data (n = 0, no model had all samples with only copy number data). Comparing the assignments done with copy number and expression to the ones using only one type of data, there were no models that changed when using only expression data versus using combined CNA-expression data and 12 that changed when using only copy number data versus using combined CNA-expression data (2 from IntClust 1 to IntClus 9, 1 from InClust 5 to IntClust 10, 1 from IntClust 7 to IntClust 8, 2 from IntClust 10 to IntClust 3, 1 from IntClust 10 to IntClust 4, 5 from IntClust 10 to IntClust 9).

As the PDTX cohort might not represent all types of breast cancers, we included the 1980 Metabric samples (Curtis et al., 2012) and quantile-normalized them together with the PDTX samples to obtain more accurate classifications on all methods.

Pathway activation scores. We downloaded the c6 oncogenic signatures from the Molecular Signatures Database (and incorporated an in-house PI3K/AKT/mTOR and an in-house DNA Damage Response DDR signature) and applied the GSVA package to infer sample specific pathway activation. Similarly, as the PDTX cohort might not represent all types of breast cancers, we also included the 1980 Metabric samples and quantile-normalized them together before converting the expression values into z-scores. For pathways that had a subset of genes that should be upregulated (UP) and another subset that should be downregulated (DOWN), the final score was obtained as UP-DOWN.

Heat maps and cluster analysis were performed using Euclidian distance and the Ward method.

Pearson correlations between log-intensity expression values or pathway activation scores in tumor and PDTX, different passages of the same model, PDTX and PDTCs of the same passage, mice replicates, technical replicates of the same sample and different tumors were computed.

A detailed comparison of pathway activity scores in tumor and PDTX and PDTX passages was performed fitting, to each pathway, a generalized additive model using the R package mgcv with the score of the tumor as the dependent variable and a smooth function of the score of all the PDTX passages as the independent variable. The deviance and the Spearman Correlation were computed as measures of predictive ability and reproducibility. Similar models were fit using the first PDTX passage as dependent variable and the rest of the passages as independent.

### Methylation Reduced Representation Bisulfite Sequencing Analysis

RRBS sequencing was performed using 125bp paired-end reads. Alignment was carried out using Bismark with our custom pipeline to remove mouse contamination. Only CpGs with at least 5 reads were selected for subsequent analysis. Methylation levels were obtained as the proportion of Cs in the CpG sites.

Pearson correlations between proportion of methylation in CpG sites in tumor and PDTX, different passages of the same model, PDTX and PDTCs of the same passage, mice replicates, technical replicates of the same sample and different tumors were computed. A filter of 1,000,000 reads was applied for this analysis.

For downstream analysis, a filter of 1.5 million reads was applied to select samples and for each gene promoter methylation score was computed as the mean value in the CpG sites in the region ± 2,000 around the TSS.

### Analysis of High-Throughput Drug Screening Using PDTCs

The observed response was computed as: 100 – (100 ^∗^ (intensity-negative control)/(positive control – negative control). Quality Control was performed comparing response values in plates and screenings done in similar dates. Non-parametric isotonic regression using the R function isoreg was fit to the set of technical replicates of a given drug response for a given sample. The area under the curve (AUC) was computed on the model fits using the package flux which uses the trapezoid rule. The half maximal inhibitory concentration (IC_50_) was predicted fitting a smoothing spline to the isotonic regression line. As a measure of variability around the AUC and IC_50_ estimates, isotonic regression curves were fit to each of the technical replicates individually and their AUC and IC_50_ also computed, producing error bars for the overall estimates based on the standard deviation. In order to compare IC_50_ estimates from drugs with different range of doses, we computed the IC_50_ as a percentage dose using the formula 100 ^∗^ (IC_50_ – minimum dose tested) / (range of doses tested).

Unsupervised clustering using Euclidean distance and the Ward method on the fitted dose-response curves showed eight different patterns that corresponded to different expected responses. We then established eight theoretical curves based on the percentage of cells dead at the five dosage points:

• (0, 0, 0, 0, 0)
• (0, 0, 0, 0, 50)
• (0, 0, 0, 50, 90)
• (0, 0, 30, 50, 90)
• (0, 25, 50, 75, 100)
• (0, 50, 60, 80, 100)
• (60, 60, 60, 60, 60)
• (100, 100, 100, 100, 100)

These curves cover increasingly sensitive response patterns, from no response to full toxicity. Each response was classified into these theoretical curves by a minimum squares approach.

Pearson correlation between technical replicates was computed on the drug responses (AUC) of the same sample and the same drug. Pearson correlation between biological replicates was computed on the drug responses (AUC) of the same model and the same drug.

Data from drug combinations was subject to the same normalization as in the single drug pipeline. As each combination screening contained also two single drug responses (for each drug in the combination), the Pearson correlation was computed comparing these and the single drug screen (previously obtained) for the same model/drug. Isotonic regression curves and AUC were fitted to the single response curves of the drugs in the combination. Synergistic effects were measured using the Bliss model (Bliss, 1939, Greco et al., 1995), which compares the observed response under a given combination of two drugs and the expected response under a model of independence. That is, if the proportion of cells dead with a dose i of a drug X is rx(i) and the proportion of cells dead with a dose j of a drug Y is rY(j), the expected proportion of cells dead under a combination of i and j is rx(i) + rY(j) - rx(i) rY(j). These values were computed by fitting an isotonic regression on each single drug response and compared with a bivariate isotonic fit on the drug combination obtained with the R package isotonic.pen. The residuals of the Bliss model were defined as the difference between the observed and the expected response. These values were taken merely as descriptive markers and no inferences were performed on them.

### Data and Software Availability

#### Software

Custom scripts to run the analyses are available at figshare: https://figshare.com/s/4a3f6bc543e5ba85834c

#### Data Resources

Raw sequencing and microarray files are available at EGA: EGAS00001001913.

Normalized/Summarized data files are available at figshare: https://figshare.com/s/4a3f6bc543e5ba85834c. This repository also contains files with a comprehensive collection of plots including Pearson and Spearman correlation of SNVs, copy number, gene expression, pathway activation and methylation, pairwise scatterplots showing variant allele frequencies in each model, mutational profiles in each sample, copy number plots for each of the samples, pairwise scatterplots showing pathway scores in each model, pairwise smoothed scatterplots showing CpG methylation scores in each model, AUC and iC50 scores for all drugs in each model tested, dose-response curves for each drug and model tested, pairwise scatterplots showing AUC scores for drugs targeting the same pathway, boxplots showing Spearman correlation between cancer pathway activity score and AUCs for compounds targeting the same pathway and results of the Bliss model for each drug combination applied to each model.

The dataset is also available in an interactive web portal that allows exploration, plotting and data downloading: http://caldaslab.cruk.cam.ac.uk/bcape

## Author Contributions

Conceptualization, A. Bruna, O.M.R., M.J.G., and C.C.; Methodology, A. Bruna, W.G., A.S.B., J.S., J.W.C., A.T.-V., A.M.-D., A. Barthorpe, H.L., M.J.O., E.M., A.L.W., V.S., S.A., M.J.G., and C.C.; Software, O.M.R., M.C., R.N.B., K.P., P.E., and M.E.; Validation, A. Bruna, O.M.R., M.J.G., and C.C.; Formal Analysis, O.M.R., M.C., R.N.B., K.P., P.E., and S.A.; Investigation, A. Bruna, W.G., A.S.B., J.S., J.W.C., A.T.-V., J.H., A.M.-D., A. Barthorpe, H.L., M.J.O., and V.S.; Resources, S.-J.S., L.J., E.P., R.B., J.G., J.C., J.B., E.M., A.L.W., and V.S.; Data Curation, A. Bruna, O.M.R., W.G., A.S.B., M.J.G., and C.C.; Writing–Original Draft, A. Bruna and C.C.; Writing–Review & Editing, A. Bruna, O.M.R., P.E., S.A., M.J.G., and C.C.; Visualization, A. Bruna, O.M.R., M.E., and C.C.; Supervision, A. Bruna, O.M.R., M.J.G., and C.C.; Project Administration, A. Bruna, O.M.R., M.J.G., and C.C.; Funding Acquisition, C.C.

## Supplemental Information

Table S1. Clinical Information of Tumor Samples Used to Originate the Breast Cancer Living Biobank, Related to Figure 1 Characteristics of established PDTX models. Stratification of PDTX models into Integrative Clusters and their ER/PR/HER2/BRCA1 status. Table S1. Clinical Information of Tumor Samples Used to Originate the Breast Cancer Living Biobank, Related to Figure 1

Table S2. Immunohistochemistry on Tissue Microarrays of PDTXs and Originating Cancers, when Available, Related to the main text Table S2. Immunohistochemistry on Tissue Microarrays of PDTXs and Originating Cancers, when Available, Related to the main text

Table S3. SNVs Grouped by Clonal Clusters and Their Cellular Prevalence as Estimated by PyClone, Related to Figure 3 and STAR Methods Table S3. SNVs Grouped by Clonal Clusters and Their Cellular Prevalence as Estimated by PyClone, Related to Figure 3 and STAR Methods

Table S4. Information on the Compound Library Used, Related to Figure 4 Table S4. Information on the Compound Library Used, Related to Figure 4

Table S5. Table Showing Information on Drug Responses in PDTC Models Using CTG, CyQUANT, and SYTOX Viability Readouts in Parallel, Related to Figure 4 and STAR Methods Table S5. Table Showing Information on Drug Responses in PDTC Models Using CTG, CyQUANT, and SYTOX Viability Readouts in Parallel, Related to Figure 4 and STAR Methods

Table S6. Information on Models and Drug Responses to PI3K, BET, and PARP Inhibitors Used in this Study, Related to Figure 4 Table S6. Information on Models and Drug Responses to PI3K, BET, and PARP Inhibitors Used in this Study, Related to Figure 4

Table S7. Table Showing Information on Ex Vivo and In Vivo Drug Response Validations, Related to Figure 6 Table S7. Table Showing Information on Ex Vivo and In Vivo Drug Response Validations, Related to Figure 6
